# Responding to Vaccine Safety Signals during Pandemic Influenza: A Modeling Study

**DOI:** 10.1371/journal.pone.0115553

**Published:** 2014-12-23

**Authors:** Judith C. Maro, Dennis G. Fryback, Tracy A. Lieu, Grace M. Lee, David B. Martin

**Affiliations:** 1 Department of Population Medicine, Harvard Medical School and Harvard Pilgrim Health Care Institute, Boston, Massachusetts, United States of America; 2 Department of Population Health Sciences, University of Wisconsin-Madison School of Medicine and Public Health, Madison, Wisconsin, United States of America; 3 Division of Research, Kaiser Permanente Northern California, Oakland, California, United States of America; 4 Center for Biologics Evaluation and Research, Food and Drug Administration, Silver Spring, Maryland, United States of America; Melbourne School of Population Health, Australia

## Abstract

**Background:**

Managing emerging vaccine safety signals during an influenza pandemic is challenging. Federal regulators must balance vaccine risks against benefits while maintaining public confidence in the public health system.

**Methods:**

We developed a multi-criteria decision analysis model to explore regulatory decision-making in the context of emerging vaccine safety signals during a pandemic. We simulated vaccine safety surveillance system capabilities and used an age-structured compartmental model to develop potential pandemic scenarios. We used an expert-derived multi-attribute utility function to evaluate potential regulatory responses by combining four outcome measures into a single measure of interest: 1) expected vaccination benefit from averted influenza; 2) expected vaccination risk from vaccine-associated febrile seizures; 3) expected vaccination risk from vaccine-associated Guillain-Barre Syndrome; and 4) expected change in vaccine-seeking behavior in future influenza seasons.

**Results:**

Over multiple scenarios, risk communication, with or without suspension of vaccination of high-risk persons, were the consistently preferred regulatory responses over no action or general suspension when safety signals were detected during a pandemic influenza. On average, the expert panel valued near-term vaccine-related outcomes relative to long-term projected outcomes by 3∶1. However, when decision-makers had minimal ability to influence near-term outcomes, the response was selected primarily by projected impacts on future vaccine-seeking behavior.

**Conclusions:**

The selected regulatory response depends on how quickly a vaccine safety signal is identified relative to the peak of the pandemic and the initiation of vaccination. Our analysis suggested two areas for future investment: efforts to improve the size and timeliness of the surveillance system and behavioral research to understand changes in vaccine-seeking behavior.

## Introduction

Responding to influenza vaccine safety signals experienced during a pandemic is a scientific and public policy challenge. Not only must federal decision-makers balance the immediate consequences of pandemic disease against uncertain vaccine risks, they also must weigh how federal actions might affect future vaccine-seeking behavior. For instance, in 1976, after initiating a National Influenza Immunization Program in response to a localized swine flu outbreak, federal authorities suspended vaccination after ten weeks because preliminary surveillance suggested that the incidence of Guillain-Barre Syndrome was approximately seven-fold greater among vaccinees [1].

Given that this particular swine flu virus was never isolated outside of Fort Dix [2], the benefit-risk calculus appears simple in hindsight. However, the decision to initiate and then withdraw a mass vaccination campaign was regarded by some as a public health failure [3], resulting in sustained and unforeseen consequences on vaccine-seeking behavior, and loss of public confidence in decision-making. Firsthand accounts [4]–[8] and historical assessments [9], [10] have emphasized the difficulty of compressed decision-making under conditions of uncertainty. While improvements in near real-time vaccine safety surveillance now allow earlier detection of vaccine safety signals [11], [12], the need to act in the context of scientific uncertainty has not changed.

These circumstances are ripe for simulation and decision models. Recent pandemic threats and the pandemic potential of H5N1 and H7N9 viruses have stimulated multiple preparedness efforts [13]–[15] including scenario-based mathematical modeling [16], [17]. Prior models have focused on influenza transmission [18], [19], optimal vaccine allocation [20]–[26], social distancing [27]–[29], antivirals [30], and layered interventions [31]–[33]. However, none have considered regulatory responses to vaccine safety signals emerging during the course of a mass vaccination program.

We addressed this gap using a multi-criteria decision analysis (MCDA) that explored regulatory decision-making in the context of emerging vaccine safety signals experienced during pandemic influenza. Specifically, the MCDA we developed evaluates the effect of several alternative regulatory responses on the transmissibility and severity of the pandemic, the burden of adverse events, and the potential for sustained changes in vaccine-seeking behavior.

## Materials and Methods

### Overview

The MCDA included several linked models. First, the vaccine safety signal was simulated in a model of the surveillance system. Next, this signal and four potential regulatory responses were the triggering input in a pandemic influenza transmission model. Each response affected short-term vaccine-associated benefits and risks (i.e., within the pandemic period), and future vaccine-seeking behavior. The outputs of the influenza transmission model were inputs to an expert-derived multi-attribute utility function. The multi-attribute utility function is used to weight and combine multiple outcomes into a single figure of merit whose expected value was maximized to select the preferred regulatory decision.

### Model of Influenza Vaccination Surveillance System

We simulated surveillance in the Post-licensure Rapid Immunization Safety Monitoring (PRISM) system, which is currently being tested for influenza vaccine safety surveillance [34]. We projected 4.3 million adopters of influenza vaccination in the PRISM system based on prior data. The methodology for the simulation model is described elsewhere [35] and a detailed description of the model is in S1 File.

#### Strength of Vaccine Safety Signals

In each scenario, we evaluated three vaccine safety signals based on historical precedent: 1) vaccine-associated febrile seizures [36]–[38], 2) vaccine-associated Guillain-Barre Syndrome [39], [40], and 3) both febrile seizures and Guillain-Barre Syndrome. We simulated a vaccine-associated febrile seizures effect size as an incidence risk difference of ∼150 excess febrile seizures per 100,000 doses in a cohort of 0–5 year olds when compared with a historical cohort of seasonal influenza vaccinees. We simulated a vaccine-associated Guillain-Barre Syndrome effect size as an incidence risk difference of 40 excess cases of Guillain-Barre Syndrome per one million doses when compared with a historical cohort of seasonal influenza vaccinees. We chose these levels of risk because they might plausibly have escaped detection in clinical trials and thus pose a particular challenge for decision-makers. We also presumed that vaccine safety signals were not unique to a single manufacturer or to a specific lot or batch number.

### Influenza Transmission Model with Bass Diffusion of Influenza Vaccine

We adapted an age-structured disease transmission model [24] and added an influenza vaccination adoption function modeled as a Bass diffusion process while assuming a universal vaccination policy [41]. Bass diffusion models are commonly used in the marketing literature to describe the diffusion of innovations [42], [43]. A detailed description of the deterministic, compartmental Susceptible-Exposed-Infectious-Recovered (SEIR) influenza transmission model and Bass diffusion process are included in S1 File.

#### Pandemic Influenza Parameters

We characterized influenza epidemics by their transmissibility, severity, and timing. We used R_0_, the basic reproduction number, to characterize transmissibility. Severity was measured by influenza morbidity and mortality. Timing refers to the amount of circulating virus present at the time vaccination began in the modeled U.S. population. Modeled circulating virus determined whether the peak of vaccination coverage was likely to precede, run concurrent with, or follow the peak of influenza transmission.

We implemented two scenarios: mild and severe influenza. The “mild” scenario was an influenza that had low transmissibility (i.e., R_0_ = 1.4), low severity, and the peak of vaccination preceded the peak of influenza transmission. The “severe” scenario involved high transmissibility (i.e., R_0_ = 2.0), moderate-to-high severity, and influenza transmission and vaccination peaked concurrently.

#### Influenza Vaccine Parameters

We assumed vaccination began September 1. We chose vaccination effectiveness parameters and expected vaccination coverage based on data observed during the H1N1 pandemic. Parameter details are in S1 File.

### Multi-Criteria Decision Analysis Model

#### Simulated Regulatory Responses

When a vaccine safety signal was detected, the MCDA was evaluated using four simulated regulatory responses: 1) *No Action* – no communication from the regulatory agency to the public; 2) *Risk Communication Alone* – risk communication issued (e.g., a “Dear Healthcare Provider” letter or website announcement) that described the vaccine safety signal and identified “at-risk” individuals for a vaccine-associated adverse event but did not recommend changes in vaccine use; 3) *Selective Suspension* – risk communication issued and vaccination suspended in “at-risk” individuals; and 4) *General Suspension* – risk communication issued and vaccination suspended for all individuals. These four responses were an informative sample as the complete range of responses was beyond the scope of this activity.

The No Action response did not alter vaccine-seeking behavior and universal vaccination coverage continued as it had before. The Risk Communication Alone response reduced vaccine-seeking behavior among the “at risk” individuals targeted in the risk communication. “At risk” individuals were defined as children age 5 and below for febrile seizures and adults age 50 and above for Guillain-Barre Syndrome based on historical data associated with these vaccine safety signals [36]–[40]. The individuals who were not at highest risk for the adverse event also reduced their vaccine-seeking behavior by a minor amount during the pandemic influenza. The Selected Suspension response reduced vaccine-seeking behavior to zero in “at risk” individuals, and all others reduced their vaccine-seeking behavior by a minor amount during the pandemic influenza. The General Suspension response reduced vaccine-seeking behavior to zero because the vaccine became unavailable.

#### Expert-Derived Multi-Attribute Utility Function

In MCDAs, decisions are characterized by multiple competing criteria, and decision-makers must consider all criteria when evaluating possible decision options. A multi-attribute utility function is a mathematical equation used to characterize the overall value (or “utility”) of each decision option relative to the others based on the specified criteria [44]. We developed an expert-derived additive multi-attribute utility function to evaluate the MCDA. The four criteria of interest were: 1) expected vaccination benefit from averted influenza as measured by a composite index of influenza cases, hospitalizations, and deaths averted; 2) expected vaccination risk from vaccine-associated febrile seizures as measured by attributable cases; 3) expected vaccination risk from vaccine-associated Guillain-Barre Syndrome as measured by attributable cases; and 4) expected change in vaccine-seeking behavior in *future* seasons as a consequence of public reaction to changes in federal vaccination policy during the pandemic. The first three criteria were directly calculated from the influenza transmission model and were limited to the pandemic time period. The fourth criterion describes vaccine-seeking behavior in future seasons. For this criterion, we used a qualitative variable with three levels: a) no change in future vaccine-seeking behavior, b) minor change: anticipated 10% reduction in future vaccine-seeking behavior, and c) major change: anticipated 25% reduction in future vaccine-seeking behavior. These levels were based on anecdotes related to the 1976 swine flu experience [4]–[8] and small studies showing that perceptions about vaccine-associated adverse events can meaningfully reduce vaccine-seeking behavior [45]–[48], but they were not validated by empirical research.

We convened an expert panel of six physicians who were currently serving, or had previously served, on vaccine-related federal advisory committees to elicit expert preferences [44], [49], [50] on prioritization of the four criteria of interest as shown in Table 1. We performed separate elicitations for the mild and severe scenarios, thereby creating separate multi-attribute utility functions for the two situations. We combined each expert panelist's multi-attribute utility function to derive the “average” decision-maker. A detailed description of the multi-attribute utility function elicitation process and result is in S1 File.

**Table 1:** Averaged Scaling Constants of the Expert Panel for Multi-Criteria Decision Analysis.

Criteria	Expected Vaccination Benefit	Expected Vaccine-associated risk from Febrile Seizures	Expected Vaccine-associated risk from Guillain-Barre Syndrome	Expected Future Change in Vaccine-Seeking Behavior
Mild Scenario^a^	0.55	0.01	0.16	0.28
Severe Scenario^b^	0.664	0.012	0.074	0.250

A scaling constant represents the relative weight given to each criterion in the utility function. Each row must sum to 1.

a The mild scenario was characterized by low transmissibility, low severity, and the peak of vaccination preceded the peak of influenza transmission.

b The severe scenario was characterized by high transmissibility, moderate-to-high severity, and the peak of vaccination occurred concurrently with the peak of influenza transmission.

#### Conditional Probability Elicitation

In the MCDA, we were interested in modeling the general public's reaction to federal vaccination policy changes and how that reaction translated into vaccine-seeking behavior in future seasons. We lacked any public preference surveys similar to those described in [51], [52] and therefore, we asked the expert panel to hypothesize about changes to future vaccine-seeking behavior as a result of reaction to federal vaccination policy. For example, we asked panelists to describe the probability that the public would a) not change their future vaccine-seeking behavior, b) reduce it by 10%, or c) reduce it by 25%, if the government received a vaccine safety signal in the mild scenario and responded with No Action. We repeated this procedure for both scenarios and all four regulatory responses. Table 2 lists their averaged probabilities.

**Table 2:** Anticipated Changes in Vaccine-Seeking Behavior associated with Four Regulatory Responses.

	Reduction in Vaccine Seeking Behavior		
	No Change (0%)	Minor Change (−10%)	Major Change (−25%)
Mild Scenario			
No Action	0.483	0.133	0.383
Risk Communication Alone	0.473	0.227	0.300
Selective Suspension	0.410	0.353	0.237
General Suspension	0.350	0.183	0.467
Severe Scenario			
No Action	0.533	0.083	0.383
Risk Communication Alone	0.590	0.190	0.220
Selective Suspension	0.517	0.257	0.227
General Suspension	0.573	0.243	0.183

This criterion, defined with the three levels in the table, links regulatory responses to vaccine-seeking behavior in the long-term. If a particular regulatory response is selected in the model, then each row represents the probability of the three levels (i.e., note that each row sums to 1.0). Therefore, regulatory responses with the highest probability of no change are associated with the highest levels of future vaccine-seeking behavior.

All modeling and subsequent analyses were completed using MATLAB and R.

## Results

### Vaccine Safety Signal Detection

**Figure 1:**
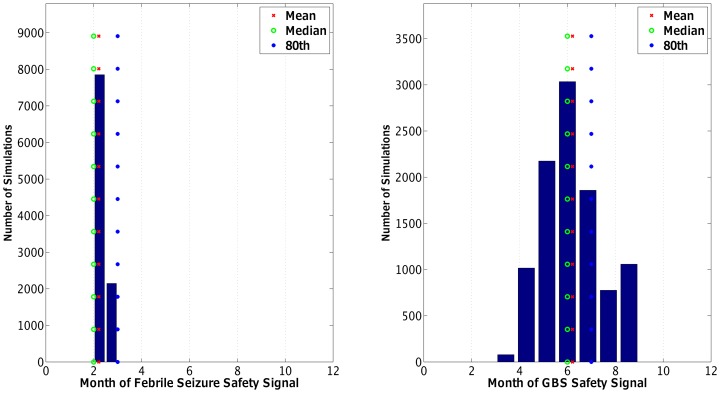
Chance of a Vaccine Safety Signal Being Detected over 10,000 Simulations. Left panel is febrile seizures; right panel is Guillain-Barre Syndrome. For Guillain-Barre Syndrome, the safety signal remains undetected (is missed) 10% of the time. “o” is the median, “x” is the mean, “*” is the 80th percentile. Other details in S1 File.

Based on our assumptions of the surveillance system, the febrile seizures signal was detected most often two months after the start of vaccination whereas the Guillain-Barre Syndrome signal was detected most often six months after the start of vaccination (Fig. 1). Statistical power to detect a vaccine safety signal at the effect size we tested was nearly 100% for febrile seizures and 90% for Guillain-Barre Syndrome. That is, in 10% of the simulations, the increased risk of vaccine-associated Guillain-Barre Syndrome was missed.

### Multi-Criteria Decision Analysis Results

**Figure 2:**
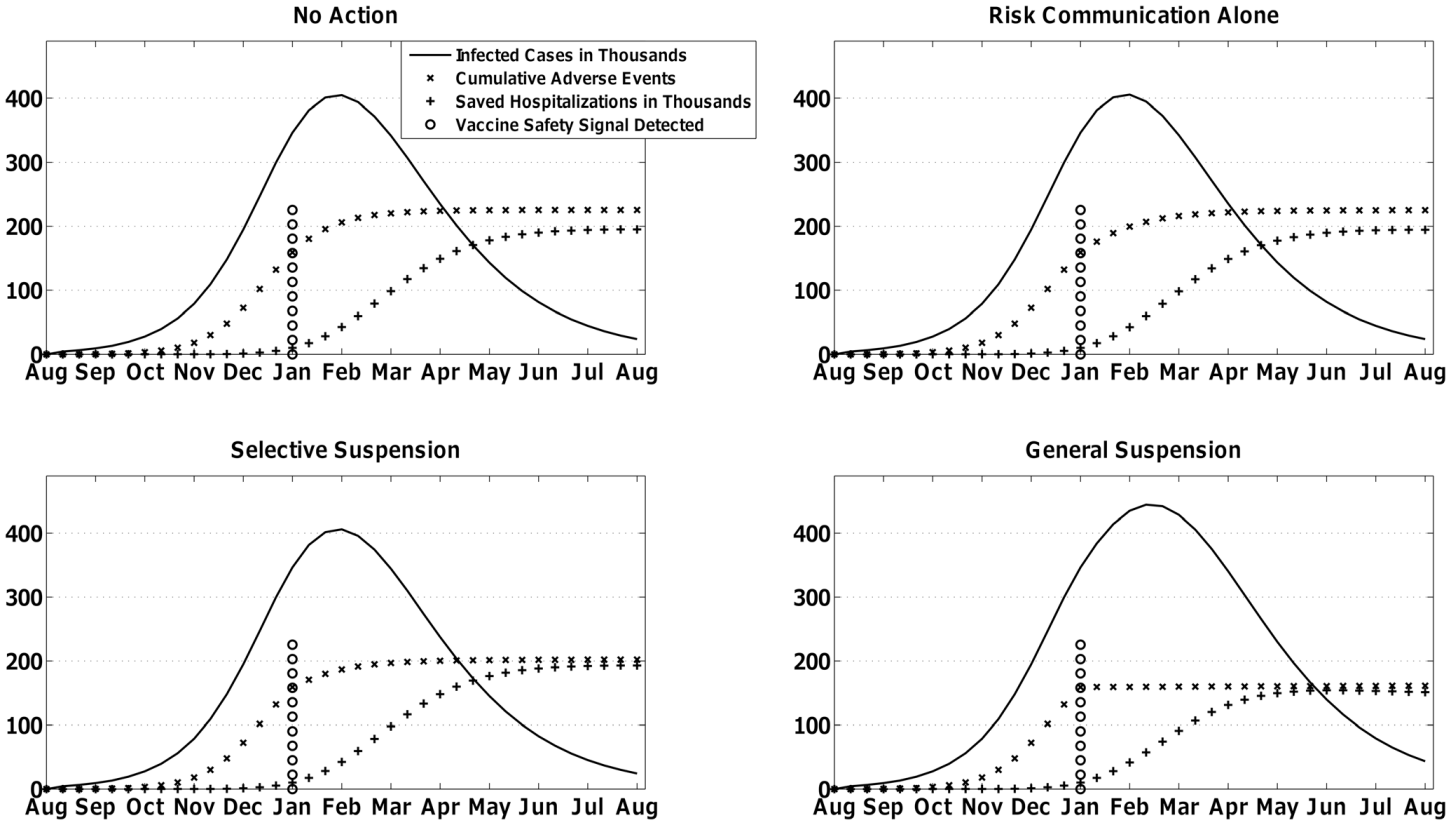
One simulation run of the mild scenario with a Guillain-Barre Syndrome safety signal received four months after the start of the vaccination campaign, i.e., January, which is five months after the start of the influenza pandemic scenario. The Multi-Criteria Decision Analysis model selected Risk Communication Alone or Selective Suspension as the preferred regulatory response.

**Figure 3:**
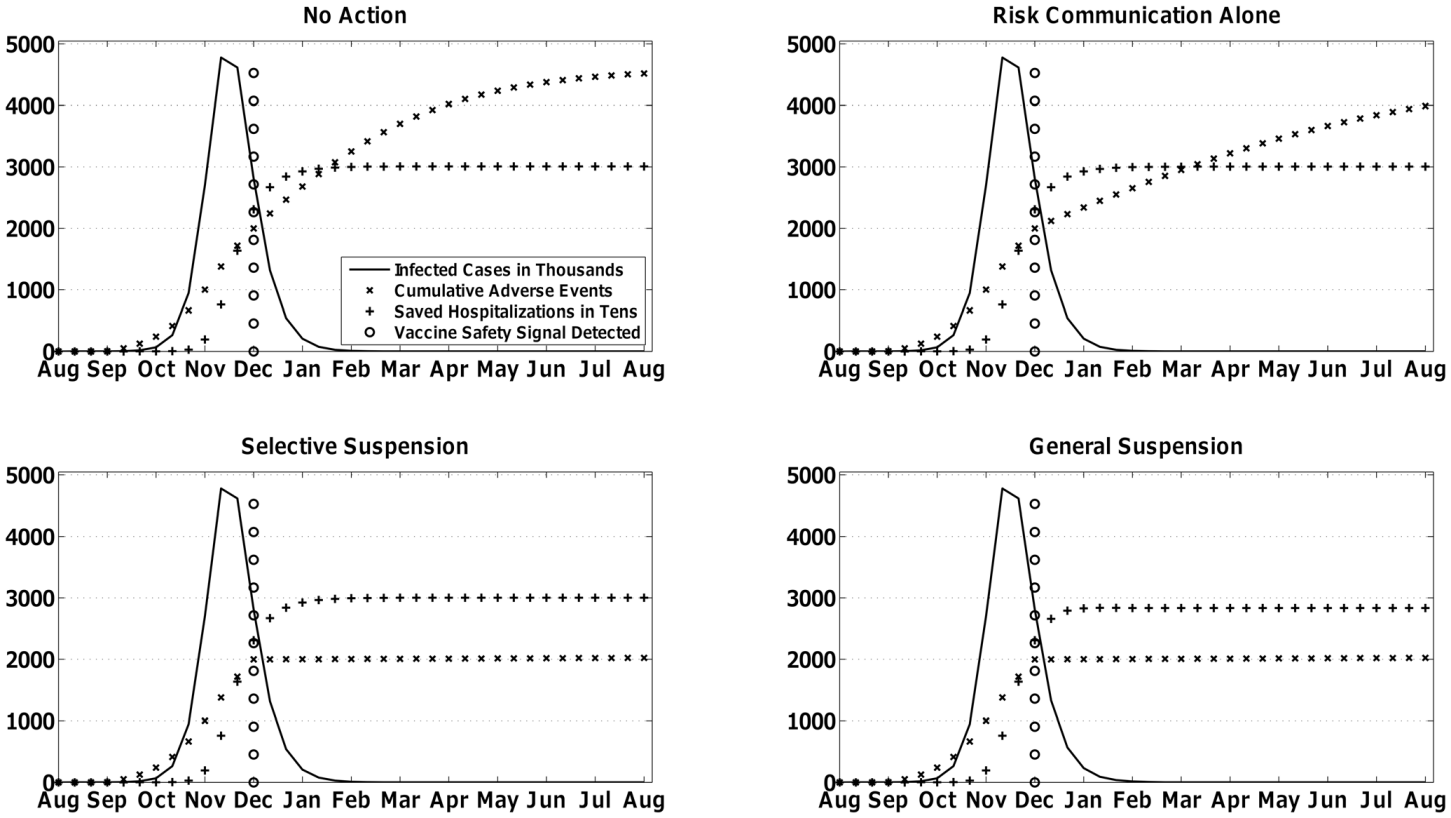
One simulation run of the severe scenario with a febrile seizures safety signal received three months after the start of the vaccination campaign, i.e., December, which is four months after the start of the influenza pandemic scenario. The outcomes that occur with each of the four simulated regulatory responses are shown in each panel. The Multi-Criteria Decision Analysis model selected Risk Communication Alone as the preferred regulatory response.

The MCDA selected the regulatory responses shown in Table 3 primarily based on the prioritization, or relative weight, assigned to the four competing criteria by the expert panel in Table 1. In both scenarios, the average decision-maker valued near-term vaccine-related outcomes relative to long-term, projected outcomes by 3∶1. Table 2 shows how the expert panel linked regulatory responses to those long-term projections. In particular, General Suspension maximized negative long-term impacts on vaccine-seeking behavior in the mild scenario whereas No Action did in the severe scenario. Figs. 2 and 3 are representative instantiations of the mild and severe scenario respectively and show the near-term vaccine-related outcomes associated with each regulatory response. Interpreted together, outcomes in Figs. 2 and 3 are modified by the weight assigned to them in Table 1 and the probability of undesirable long-term effects in Table 2. Fig. 1 represents the chance of vaccine safety signal detection in the months following the start of vaccination.

**Table 3:** Multi-Criteria Decision Analysis Results.

Regulatory Response Selected by the Multi-Criteria Decision Analysis Model										
	Month After the Start of the Vaccination Campaign that Vaccine Safety Signal was Received									
	2 (NOV)		3 (DEC)		4 (JAN)		5 (FEB)		6 (MAR)	
Mild Scenario										
Febrile Seizures Signal Only	Risk Communication/Selective Suspension		Risk Communication/Selective Suspension		NA		NA		NA	
GBS Signal Only	NA		Selective Suspension		Risk Communication/Selective Suspension		Risk Communication/Selective Suspension		Risk Communication/Selective Suspension	
Febrile Seizures and GBS Signals	NA		Selective Suspension		Risk Communication/Selective Suspension		Risk Communication/Selective Suspension		Risk Communication/Selective Suspension	
Severe Scenario										
Febrile Seizures Signal Only	Risk Communication		Risk Communication		NA		NA		NA	
GBS Signal Only	NA		Risk Communication		Risk Communication/Selective Suspension/General Suspension		Risk Communication/General Suspension		Risk Communication	
Febrile Seizures and GBS Signals	NA		Risk Communication		Risk Communication/Selective Suspension/General Suspension		Risk Communication/General Suspension		Risk Communication	

The chance of a vaccine safety signal being detected in a given month is shown in Fig. 1. NA implies that the probability is zero. In the febrile seizures case, this occurred because the surveillance system is powered to detect the signal 2–3 months after the start of vaccination.

Abbreviations: GBS, Guillain-Barre Syndrome; NA, not applicable.

#### Mild Scenario

In the mild scenario, by the time intervention opportunities occurred with a probability described in Fig. 1, the peak of vaccination had passed and the indirect benefits of vaccination had been achieved. Therefore, vaccine associated-benefits for all regulatory responses except for General Suspension were roughly similar. Over the multiple intervention opportunities and types of vaccine safety signals in Table 3, General Suspension was not preferred because of its lower vaccine-related benefit (see Fig. 2). (Note: the MCDA used a composite index for benefits but we show saved hospitalizations here.) Also, No Action was not preferred because of its projected impact on future vaccine-seeking behavior as described in Table 2. With those regulatory responses eliminated, the MCDA was indifferent between the remaining two options.

Generally, Risk Communication Alone had marginally higher vaccine-associated benefit than Selective Suspension (e.g., a difference of ∼2000 saved hospitalizations in Fig. 2), but also had higher vaccine-associated risk (e.g., a difference of 24 Guillain-Barre Syndrome cases in Fig. 2).

#### Severe Scenario

In the severe scenario, intervention opportunities occurred with the same probability as in the mild scenario, but the timing of peak vaccination coincided with peak influenza transmission, which meant that decision-makers had almost no influence on the pandemic's near-term outcomes (see Fig. 3). With near-term vaccine-related benefits converging for the four regulatory responses, the MCDA was driven by projected long-term impacts on future vaccine-seeking behavior. In other words, with little leverage in the present pandemic, decision-makers focused on the future. Under these circumstances, the MCDA favored Risk Communication Alone most consistently over multiple intervention points in Table 3 because it minimized negative impacts on future vaccine-seeking behavior as shown in Table 2.

Depending on the month of detection of the vaccine safety signal, the MCDA was sometimes indifferent between Risk Communication Alone and other options when a Guillain-Barre Syndrome safety signal was detected. This occurred because avoided vaccine-associated Guillain-Barre Syndrome cases compensated for greater negative impacts on future vaccine-seeking behavior. However, the number of vaccine-associated Guillain-Barre Syndrome cases that offset the projected negative impact was quite low (e.g. 5 avoided cases). Compensation sufficient to alter the MCDA's calculated preferences did not occur with a febrile seizures signal because of the low weight placed on avoided vaccine-associated febrile seizures.

### Sensitivity Analyses

#### Timing of Vaccination Availability

We varied the availability of vaccination from August 1 (day 1) to November 1 (day 90). In the mild scenario, the decision was unchanged from the results shown in Table 3, but the logic contributing to the decision did change. In cases when the timing of vaccine safety signal detection preceded the peak of the influenza epidemic (February 1), the MCDA was indifferent between Risk Communication Alone and Selective Suspension because the former was associated with greater vaccine-associated benefits and greater vaccine-associated risk. However, when vaccine safety signal detection aligned with the peak of the influenza pandemic, then vaccine-associated benefits and risks converged among decision options, and the projected impact on future vaccine-seeking behavior determined the decision. Unlike in the severe scenario when Risk Communication Alone has the best projection on this attribute, in the mild scenario, Risk Communication Alone and Selective Suspension are nearly tied on this attribute and the MCDA is indifferent among them for this reason.

In the severe scenario, if vaccine is available August 1 at the start of the pandemic scenario and thus precedes the peak of influenza, Risk Communication Alone is the preferred decision option for any receipt of a vaccine safety signal. It performs the best on vaccination-related benefits and projected impacts on future vaccine-seeking behavior.

#### Expert Preferences

We recalibrated the multi-attribute utility function with equal prioritization for the four criteria in Table 1 (i.e., all set to 0.25) and re-ran the mild scenario. Here, vaccine-associated risk was more important to the decision-maker since its weight increased from 0.01 to 0.25 for vaccine-associated febrile seizures and 0.16 to 0.25 for vaccine-associated Guillain-Barre Syndrome. However, these adjustments did not change the projected impact on future vaccine-seeking behavior (i.e., the probabilities in Table 2), and No Action and General Suspension remained undesirable. The MCDA consistently preferred Selective Suspension to Risk Communication Alone regardless of when the safety signal was received because more weight was assigned to avoiding excess vaccine-related risk.

#### Vaccine Effectiveness

We also evaluated the MCDA while re-parameterizing the vaccine to be half as effective. While absolute levels of vaccine associated-risks and benefits changed, these changes did not affect the relative standing among regulatory responses. Therefore, the decision was insensitive to the changed parameters.

## Discussion

We developed an MCDA to evaluate regulatory decision-making following the emergence of vaccine safety signals and evaluated potential regulatory responses. The MCDA selected the best *relative* regulatory response among available choices according to the averaged, expert-elicited preferences by maximizing the expected value of the utility of these options. Numerically, the expected values were often close among options, even though these regulatory responses carry different logistical, social, and risk communication implications. Over multiple safety signal timing situations, Risk Communication Alone was consistently the preferred option in both scenarios, closely followed by Selective Suspension in the mild pandemic scenario cases only. The preferred decision might change if the multi-attribute utility function were developed with other stakeholders' preferences.

### Why a Multi-Criteria Decision Analysis Is Important

The MCDA prompts decision-makers to transparently and explicitly describe the determinants of their decision-making by weighting multiple competing criteria. While we present two scenarios herein, the model is flexible enough to be re-run quickly with multiple sets of differing assumptions to understand which regulatory responses are robust under numerous circumstances.

Other regulatory agencies have vetted the use of MCDA to aid decision-making [53]. The general structure of our pandemic influenza MCDA enables regulatory decision-makers outside of the U.S. to utilize our approach. However, sub-model components would benefit from enhanced specificity for extension beyond the U.S. For instance, we chose simulated regulatory responses that are globally generalizable, but other users might want to precisely tailor the responses to their country or region. Also, we modeled detection of vaccine safety signals in the U.S. PRISM medical product safety surveillance system, but other regional medical product safety surveillance systems would need to be explicitly modeled to determine their performance in a pandemic context. The multi-attribute utility function could be adopted, but it could also be re-parameterized using input from experts in other countries and regions.

### What This Multi-Criteria Decision Analysis Tells Us

When the near-term benefits and risks of regulatory responses converge, the MCDA highlighted the weight decision-makers gave to the long-term stability of vaccination programs. That is, Risk Communication Alone and Selective Suspension were more desirable because of perceptions that No Action or General Suspension would have created undesirable effects on future vaccine-seeking behavior. Timing was critical to the MCDA, particularly the temporal relationships between the influenza epidemic peak, initiation of vaccination, and detection of the vaccine safety signal. These temporal relationships were most directly affected by the availability and adoption of vaccine. For example, early cycle vaccinations (i.e., September or October) had a greater per-vaccination ability to retard influenza transmission than late-cycle vaccinations (i.e., February or March) because there was higher potential for indirect vaccine-related benefits. Also important was the vaccine-attributable risk of adverse events. If the vaccine-attributable risk were higher, then signal detection would likely occur earlier and decision-makers would have more opportunity to affect near-term (i.e., within the present pandemic) vaccine-related benefits and risks.

### Limitations of the Model

First, the weakest element of the MCDA was the mapping of simulated regulatory responses to future vaccine-seeking behavior, for which we relied on the expert panel's assessment. This element drove decision-making whenever intervention opportunities occurred too late in the pandemic to influence its near-term outcomes. Recent work by Blyth et al. [51] suggests that a major reduction in vaccine-seeking behavior following a selective suspension in a mild scenario might be more likely than the probability assigned during this exercise. Additionally, Blyth et al. 's paper suggests that a “major reduction” in vaccine-seeking behavior could be modeled as high as 35%. If the probability of a major reduction in future-vaccine seeking behavior following Selective Suspension in the mild scenario were higher, then we would expect that Selective Suspension would be a less desirable option. Consequently, we would expect Risk Communication Alone to be the preferred decision option in most mild scenarios.

While the present probabilistic assumptions about long-term vaccine-seeking behavior may be imperfect, we know decision-makers are focused on preserving the integrity of such behavior. Future studies should ascertain how vaccine safety guidance or warnings in one influenza season/pandemic affect the public's vaccine-seeking behavior during subsequent seasons.

Second, as with any modeling effort, the influenza transmission sub-model and the surveillance system sub-model required multiple assumptions during parameterization. The outputs of these models – vaccine-related outcomes and the timing of signal detection – reflected these assumptions.

Third, within the multi-attribute utility function, we created a composite index of vaccine-related benefits. Within that index, we weighted all influenza-associated deaths equally, regardless of age. In the future, it may be preferred to weight morbidity by age, or to adopt a structure analogous to cumulating quality-adjusted life years [54].

Fourth, we examined risks and benefits with respect to the entire population. Future modeling efforts could focus on risks and benefits for subpopulations such as the elderly, infants or pregnant women.

Fifth, we chose not to model brand-specific risks although vaccine safety problems in recent years have been attributed to particular products [55]. Future extensions of the model could make assumptions about the age-specific market share of various products and model a safety problem that was isolated to a particular manufacturer.

### Lessons Learned for Future Pandemic Influenza Preparedness Efforts in PRISM

Low attributable risks for rare adverse events pose a significant challenge to any medical product safety surveillance system. If PRISM can detect safety signals earlier in a pandemic, then decision-makers will have a greater impact on near-term vaccine benefits and risks. Opportunities for earlier detection necessitate increasing sample size early in a pandemic. This can be accomplished by encouraging earliest possible vaccination within a pandemic period or season, adding additional electronic healthcare databases to the PRISM system to increase the number of vaccinees under surveillance, and accessing the existing PRISM databases more frequently (e.g., using a weekly update instead of a monthly update) [56].

## Supporting Information

S1 File **Model Parameters and Description of the Age-Structured Disease Transmission Model, Vaccine Safety Surveillance System Model, and Additive Multi-Attribute Utility Function.**
(PDF)
