# Supplementary material for: Responding to Vaccine Safety Signals during Pandemic Influenza: A Modeling Study
Source: PLoS One. 2014 Dec 23;9(12):e115553. doi: 10.1371/journal.pone.0115553 (PMC4275236; doi:10.1371/journal.pone.0115553)
Supplement: S1 File — Model Parameters and Description of the Age-Structured Disease Transmission Model, Vaccine Safety Surveillance System Model, and Additive Multi-Attribute Utility Function. (PDF) [file pone.0115553.s001.pdf]

Supporting Information

1 Age-Structured Disease Transmission Model

The regulatory decision model on vaccination policy required inputs on expected vaccination, vaccination-associated benefits, and vaccination-associated risks. To model these inputs, we adapted a published, age-structured disease transmission model [1]. Specifically, we added an influenza vaccination adoption function modeled as a Bass diffusion process [2]. We used the outputs of the adapted transmission model to seed the regulatory decision model.

1.1 Transmission Model Flow and Variable Definition

We used an age-structured disease transmission model to simulate the spread of pandemic influenza. A graphical display of the flow of persons is shown in Figure 1:

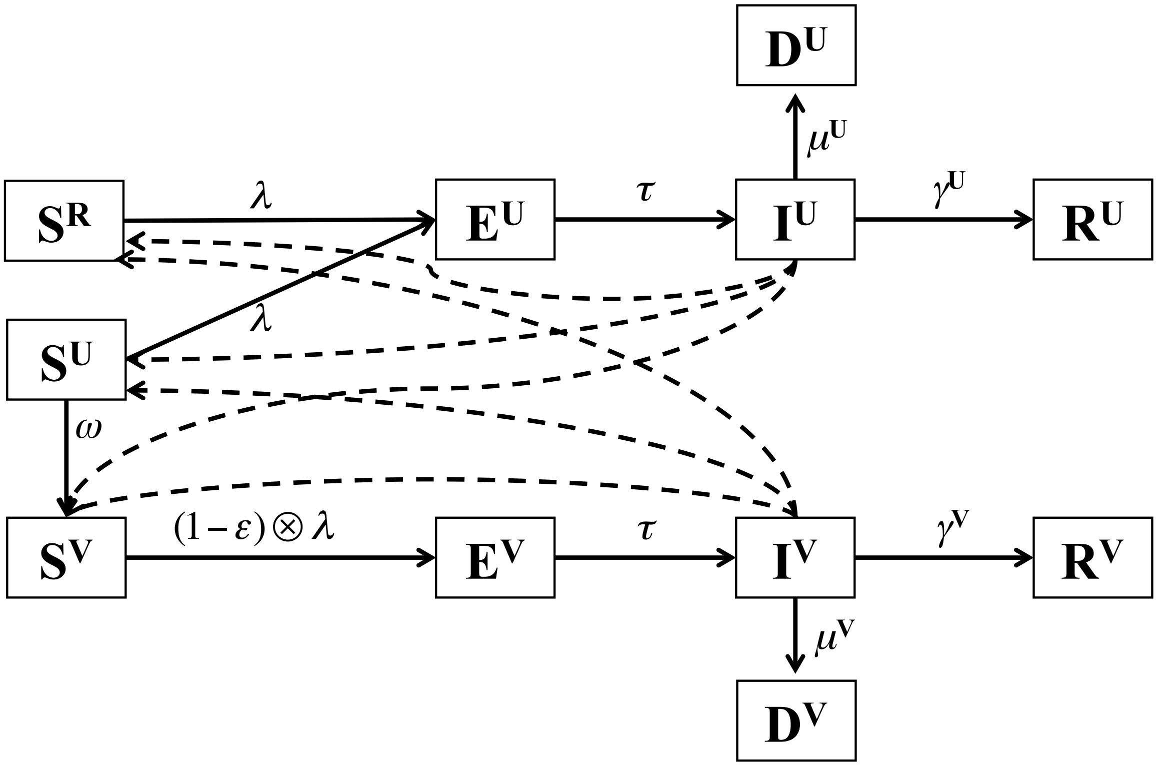

Figure 1. Influenza Transmission Flow.
Solid lines are transition paths between compartments. Dashed lines represent modes of influenza transmission. The top "track" represents unvaccinated individuals and the bottom "track" represents vaccinated individuals. The variables displayed above are written in vector notation, as each variable is a vector whose elements are age-specific.

The model variables were as follows, where the subscript *i* denotes the *i*th age group and the time unit *t* is in days:

1.1.1 Time-Dependent Variables

$S_i^R(t)$: the number of susceptible, unvaccinated individuals that were projected **not to vaccinate** in the *i*th age group at time *t*;

$S_i^U(t)$: the number of susceptible, unvaccinated individuals that were projected to vaccinate in the *i*th age group at time *t*;

$E_i^U(t)$: the number of exposed (infected), unvaccinated individuals in the *i*th age group at time *t*;

$I_i^U(t)$: the number of infectious, unvaccinated individuals in the *i*th age group at time *t*;

$R_i^U(t)$: the number of recovered, unvaccinated individuals in the *i*th age group at time *t*;

$D_i^U(t)$: the cumulative number of influenza-associated deaths among the unvaccinated population in the *i*th age group at time *t*;

$H_i^U(t)$: the cumulative number of hospitalized individuals among the unvaccinated population in the *i*th age group at time *t*;

$S_i^V(t)$: the number of susceptible, vaccinated individuals in the *i*th age group at time *t*;

$E_i^V(t)$: the number of exposed (infected), vaccinated individuals in the *i*th age group at time *t*;

$I_i^V(t)$: the number of infectious, vaccinated individuals in the *i*th age group at time *t*;

$R_i^V(t)$: the number of recovered, vaccinated individuals in the *i*th age group at time *t*;

$D_i^V(t)$: the cumulative number of influenza-associated deaths among the vaccinated population in the *i*th age group at time *t*;

$H_i^V(t)$: the cumulative number of hospitalized individuals among the vaccinated population in the *i*th age group at time *t*;

$V_i(t)$: the cumulative number of vaccinated individuals in the *i*th age group at time *t*;

$\lambda_i(t)$: the frequency-dependent influenza incidence rates for individuals in the *i*th age group at time *t*, also known as the force of infection;

$\omega_i(t)$: the conditional probability that an individual in age group *i* will vaccinate at exactly time *t* since introduction of the vaccination, given that the individual has not vaccinated before that time; also known as the hazard function;

1.1.2 Additional Variables

c_{ij}: the average daily contact rate of an individual in the *i*th age group with an individual in the *j*th age group;

β_{ij}^{U}: the per contact transmission probability of a susceptible individual in the *i*th age group by an unvaccinated infectious individual in the *j*th age group;

β_{ij}^{V}: the per contact transmission probability of a susceptible individual in the *i*th age group by a vaccinated infectious individual in the *j*th age group;

ε_i: the age-specific vaccine effectiveness against influenza infection for an individual in the *i*th age group;

ε_i^{H}: the age-specific vaccine effectiveness against influenza-associated hospitalization for an individual in the *i*th age group;

ε_i^{D}: the age-specific vaccine effectiveness against influenza-associated death for an individual in the *i*th age group;

τ_i: 1/the average incubation time for an individual in the *i*th age group;

γ_i^{U}: 1/the average duration of infectiousness for an unvaccinated individual in the *i*th age group; also known as the recovery rate;

γ_i^{V}: 1/the average duration of infectiousness for a vaccinated individual in the *i*th age group; also known as the recovery rate;

h_i^{U}: the influenza-associated hospitalization rate in hospitalizations per time *t* for an unvaccinated individual in the *i*th age group;

h_i^{V}: the influenza-associated hospitalization rate in hospitalizations per time *t* for an unvaccinated individual in the *i*th age group;

μ_i^{U}: the influenza-associated death rate in deaths per time *t* for an unvaccinated individual in the *i*th age group;

μ_i^{V}: the influenza-associated death rate in deaths per time *t* for an unvaccinated individual in the *i*th age group;

δ_i^{U}: the per capita death rate in deaths per persons for an unvaccinated individual in the *i*th age group;

δ_i^{V}: the per capita death rate in deaths per persons for a vaccinated individual in the *i*th age group;

η_i^{U}: the per capita hospitalization rate in hospitalizations per persons for an unvaccinated individual in the *i*th age group;

η_i^{V}: the per capita hospitalization rate in hospitalizations per persons for a vaccinated individual in the *i*th age group;

π_i: the proportion of infected persons in the *i*th age group, also known as the attack rate;

υ_i: the probability of vaccination in the *i*th age group.

1.2 Transmission Model Equations

1.2.1 System of Differential Equations

The model is a system of 15×*n* ordinary differential equations, with *n* age-groups and *i*=1, 2,…, *n*. The time unit *t* is in days. The model did not incorporate demographic processes because we considered the vaccination policy for a single pandemic influenza period. Additionally, the model did not incorporate concepts associated with pre-existing immunity or waning immunity. Finally, the model did not consider the effects of the use of antiviral medications or other non-pharmaceutical interventions in mitigating the influenza epidemic.

The model equations are as follows:

$$\frac{dS_i^R}{dt} = -\lambda_i(t)S_i^R(t) \tag{S1-1}$$

$$\frac{dS_i^U}{dt} = -\left[\lambda_i(t) + \omega_i(t)\right]S_i^U(t) \tag{S1-2}$$

$$\frac{dE_i^U}{dt} = \lambda_i(t)\left[S_i^R(t) + S_i^U(t)\right] - \tau E_i^U(t) \tag{S1-3}$$

$$\frac{dI_i^U}{dt} = \tau E_i^U(t) - \left[\gamma^U + \mu_i^U\right]I_i^U(t) \tag{S1-4}$$

$$\frac{dR_i^U}{dt} = \gamma^U I_i^U(t) \tag{S1-5}$$

$$\frac{dD_i^U}{dt} = \mu_i^U I_i^U(t) \tag{S1-6}$$

$$\frac{dH_i^U}{dt} = h_i^U I_i^U(t) \tag{S1-7}$$

$$\frac{dS_i^V}{dt} = \omega_i(t)S_i^U(t) - (1-\varepsilon_i)\lambda_i(t)S_i^V(t) \tag{S1-8}$$

$$\frac{dE_i^V}{dt} = (1-\varepsilon_i)\lambda_i(t)S_i^V(t) - \tau E_i^V(t) \tag{S1-9}$$

$$\frac{dI_i^V}{dt} = \tau E_i^V(t) - \left[\gamma^V + \mu_i^V\right]I_i^V(t) \tag{S1-10}$$

$$\frac{dR_i^V}{dt} = \gamma^V I_i^V(t) \tag{S1-11}$$

$$\frac{dD_i^V}{dt} = \mu_i^V I_i^V(t) \tag{S1-12}$$

$$\frac{dH_i^V}{dt} = h_i^V I_i^V(t) \tag{S1-13}$$

$$\frac{dV_i}{dt} = \frac{dS_i^V}{dt} + \frac{dE_i^V}{dt} + \frac{dI_i^V}{dt} + \frac{dR_i^V}{dt} + \frac{dD_i^V}{dt} \tag{S1-14}$$

$$\frac{d\omega_i}{dt} = \frac{q_i}{\Omega}\frac{dV_i}{dt} \tag{S1-15}$$

1.2.2 Calculation of the Force of Infection

$$\lambda_i(t) = \sum_{j=1}^{n} \frac{c_{ij}\left[\beta_{ij}^U I_j^U(t) + \beta_{ij}^V I_j^V(t)\right]}{N_i(t)} \tag{1-16}$$

where $N_i(t) = \sum_{j=1}^{n}\left[S_j^R + S_j^U + E_j^U + I_j^U + R_j^U + S_j^V + E_j^V + I_j^V + R_j^V\right]$ (1-17)

1.2.3 Calculation of Death and Hospitalization Rates

To calculate the influenza-associated death and hospitalization rates, we relied on estimation using information on the per capita death and hospitalization rates and concepts from survivability analysis [3].

$$\mu_i^U = \gamma_i^U \frac{\delta_i^U}{\left(\pi_i - \delta_i^U\right)} = \gamma_i^U \frac{\mathrm{M}_i}{1-\mathrm{M}_i} \tag{1-18}$$

$$\mu_i^V = \gamma_i^V \frac{(1-\varepsilon_i^D)\delta_i^V}{\left[\pi_i - (1-\varepsilon_i^D)\delta_i^V\right]} = \gamma_i^V \frac{(1-\varepsilon_i^D)\mathrm{M}_i}{1-(1-\varepsilon_i^D)\mathrm{M}_i} \tag{1-19}$$

where M_i is the familiar case fatality ratio, given as influenza cases resulting in death divided by influenza cases.

$$h_i^U = \gamma_i^U \frac{\eta_i^U}{\left(\pi_i - \eta_i^U\right)} \tag{1-20}$$

$$h_i^V = \gamma_i^V \frac{(1-\varepsilon_i^H)\eta_i^V}{\left[\pi_i - (1-\varepsilon_i^H)\eta_i^V\right]} \quad (1\text{-}21)$$

1.2.4 Vaccination Adoption Function

The influenza vaccination adoption function was modeled as a Bass diffusion process [2] given by the following equation:

$$\omega_i(t) = p_i + \frac{q_i}{\Omega} V_i(t) \quad (1\text{-}22)$$

where p_i and q_i are the Bass coefficients of innovation and imitation, respectively.
Ω is the total population of those expected to vaccinate. In Bass diffusion models, all individuals that are expected to vaccinate will do so.

$$\Omega = \bar{N} \sum_{i=1}^{n} \upsilon_i \theta_i \quad (1\text{-}23)$$

$\bar{N}$ is the total population at the start of the influenza period.
θ_i is the proportion of the total population in the *i*th age group.

1.2.5 Reproduction Number

We used the next-generation matrix technique [4,5] to derive R_0, the basic reproduction number in our model. R_0 is the average number of new (secondary) influenza cases that an index influenza case generates over the course of its infectious period. The reproduction number is the spectral radius, or the largest eigenvalue, of the "next-generation" matrix $\mathbf{FW^{-1}}$. That is, $R_0 = \rho\left(\mathbf{FW^{-1}}\right)$.

The next-generation matrix technique requires identification of *m* infected compartments, which contain x_k infected individuals where k =1, 2,…, *m*.

$$\frac{dx_k}{dt} = F_k(x) - \left[W_k^-(x) - W_k^+(x)\right] \quad (1\text{-}24)$$

$F_k(x)$: the incidence of new infections in the *k*th compartment;

$W_k(x)$: the net transfer rate of individuals in the *k*th compartment for reasons other than the incidence of infections;

We employed matrix notation to describe these variables. $\otimes$ indicates element-by-element matrix multiplication.

$$\mathbf{x} = \begin{bmatrix} \mathbf{E}_\mathrm{U} \\ \mathbf{I}_\mathrm{U} \\ \mathbf{E}_\mathrm{V} \\ \mathbf{I}_\mathrm{V} \end{bmatrix} \qquad \mathcal{F} = \begin{bmatrix} \lambda \otimes (\mathbf{S}^\mathrm{R} + \mathbf{S}^\mathrm{U}) \\ 0 \\ \lambda \otimes (1-\varepsilon) \otimes \mathbf{S}^\mathrm{V} \\ 0 \end{bmatrix} \qquad \mathcal{W} = \begin{bmatrix} \tau \otimes \mathbf{E}^\mathrm{U} \\ -\tau \otimes \mathbf{E}^\mathrm{U} + \left(\gamma^\mathrm{U} + \mu^\mathrm{U}\right) \otimes \mathbf{I}^\mathrm{U} \\ \tau \otimes \mathbf{E}^\mathrm{V} \\ -\tau \otimes \mathbf{E}^\mathrm{V} + \left(\gamma^\mathrm{V} + \mu^\mathrm{V}\right) \otimes \mathbf{I}^\mathrm{V} \end{bmatrix}$$

In our model, all individuals who were not infected at t=0 were susceptible. R_0 was calculated at time t=0. Therefore in the next-generation matrix $\mathbf{FW}^{-1}$, $\mathbf{S}^\mathbf{R}$, $\mathbf{S}^\mathrm{U}$, and $\mathbf{S}^\mathrm{V}$ were all calculated at time t=0. Because $\mathbf{FW}^{-1}$ has two rows of zero submatrices, R_0 was the spectral radius, or largest eigenvalue of the reduced $\mathbf{FW}^{-1}$, shown below.

$$\mathbf{FW}^{-1}_{\mathrm{red}} = \begin{bmatrix} \frac{1}{N}\left\{\left[(\mathbf{S}^\mathrm{U} + \mathbf{S}^\mathrm{R}) \otimes \frac{1}{\gamma^\mathrm{U} + \mu^\mathrm{U}}\right] 1^\mathrm{T}\right\} \otimes \mathbf{c} \otimes \beta^\mathrm{U} & \frac{1}{N}\left\{\left[(\mathbf{S}^\mathrm{U} + \mathbf{S}^\mathrm{R}) \otimes \frac{1}{\gamma^\mathrm{V} + \mu^\mathrm{V}}\right] 1^\mathrm{T}\right\} \otimes \mathbf{c} \otimes \beta^\mathrm{V} \\ \frac{1}{N}\left\{\left[(1-\varepsilon) \otimes \mathbf{S}^\mathrm{V} \otimes \frac{1}{\gamma^\mathrm{U} + \mu^\mathrm{U}}\right] 1^\mathrm{T}\right\} \otimes \mathbf{c} \otimes \beta^\mathrm{U} & \frac{1}{N}\left\{\left[(1-\varepsilon) \otimes \mathbf{S}^\mathrm{V} \otimes \frac{1}{\gamma^\mathrm{V} + \mu^\mathrm{V}}\right] 1^\mathrm{T}\right\} \otimes \mathbf{c} \otimes \beta^\mathrm{V} \end{bmatrix}$$

where 1=(1,1,...,1) is a vector of length n.

Algorithmically, in our model, we set R_0 at the desired level and then found β^U and β^V to achieve that level. For simplicity, we set $\beta^\mathrm{U} = \beta^\mathrm{V}$ for all age groups, which implied that the probability of transmission of influenza was independent of both age and vaccination status.

1.3 Epidemiologic Parameters of Influenza

We ran two scenarios, utilizing the framework set forth by Reed *et al.* [6] that characterized influenza pandemics by both their transmissibility and severity. The first scenario had lower transmissibility and severity, and we referred to this scenario as the "mild" scenario. The second scenario was modeled after the 1957 pandemic, which had higher transmissibility and severity than the mild scenario but was not quite as transmissible and severe as the 1918 pandemic. We referred to this second scenario as the "severe" scenario.

For all scenarios, we assumed that the duration of infectiousness, influenza hospitalization rate and influenza death rate were independent of vaccination status (i.e., an ineffective vaccination did not impart partial protection against hospitalization or death). That is, $\gamma_i^U = \gamma_i^V$, $\eta_i^U = \eta_i^V$, and $\delta_i^U = \delta_i^V$.

1.3.1 Mild Scenario

We set R_0 = 1.4 in the mild scenario [7,8].

Age Group	Incubation Time (τ_i^{-1}) (days) [7,9]	Duration of Infectiousness (γ_i^{-1}) (days) [10–14]	Influenza Attack Rate (π_i) (cases per 100 persons) [8,15,16]	[a]Per Capita Hospitalization Rate (η_i) (hospitalizations per 100,000 persons) [8]	Per Capita Death Rate (δ_i) (deaths per 100,000 persons) [8,17]
0-4	3	7	32	50.62*multiplier	0.39
5-17	3	7	32	19.74*multiplier	0.34
18-24	3	7	12	17.30*multiplier	0.48
25-49	3	7	12	13.86*multiplier	0.80
50-64	3	7	12	19.76*multiplier	1.38
65+	3	7	4	14.52*multiplier	0.87

Table 1. Epidemiological Parameter Values used to model the Mild Scenario.
[a]The per capita hospitalization rate is taken from the Aggregate Hospitalization and Deaths Reporting Activity (AHDRA), which was a special project initiated by the Centers for Disease Control and Prevention (CDC) during the pandemic. Because AHDRA numbers likely undercounted actual hospitalizations and deaths, the CDC developed a multiplier factor of 2.7 to account for underascertainment [18]. We also use the multiplier factor.

1.3.2 Severe Scenario

We set R_0 =2.0 in the severe scenario [19].

Age Group	Incubation Time (τ_i^{-1}) (days) [19]	Duration of Infectiousness (γ_i^{-1}) (days) [19]	Influenza Attack Rate (π_i) (cases per 100 persons) [20]	Per Capita Hospitalization Rate (η_i) (hospitalizations per 100,000 persons) [21]	Per Capita Death Rate[a] (δ_i) (deaths per 100,000 persons) [22]
0-1	1.9	4.1	32	42.7	11.57
2-4	1.9	4.1	32	42.7	3.08
5-9	1.9	4.1	33	7.2	1.43
10-14	1.9	4.1	52	3.5	1.43
15-19	1.9	4.1	54	2.5	2.54
20-24	1.9	4.1	27	5.2	2.66
25-34	1.9	4.1	27	8.8	2.66
35-39	1.9	4.1	25	11.6	2.66

40-44	1.9	4.1	18	11.6	2.66
45-49	1.9	4.1	17	13.0	8.87
50-54	1.9	4.1	19	13.0	8.87
55-59	1.9	4.1	17	20.7	8.87
60-64	1.9	4.1	10	20.7	8.87
65-69	1.9	4.1	10	72.5	28.2
70+	1.9	4.1	10	72.5	51.3

Table 2. Epidemiological Parameter Values used to model the Severe Scenario.
[a]The age groups used to describe influenza death rates in [22] were not quite lined up with our age groups for the very young and elderly. Therefore, we used his ">1" value for our 0-1 and his "75+" value for our 70+.

1.4 Vaccination Parameters

We estimated Bass coefficients p_i and q_i from data from the Post-Licensure Immunization Safety and Monitoring (PRISM) system [23]. See Section 3. We assumed these coefficients were reflective of the entire US population.

We assumed that vaccine effectiveness against influenza-related hospitalization and influenza-related death to be the same as vaccine effectiveness against influenza. That is, $\varepsilon_i = \varepsilon_i^H = \varepsilon_i^D$.

Age Group	Expected Vaccination Coverage (υ_i) (%) [24]	[a]Bass p_i	[a]Bass q_i	Vaccine Effectiveness against Influenza (ε_i) (%) [25]	Vaccine Effectiveness against Hospitalization (ε_i^H) (%)	Vaccine Effectiveness against Death (ε_i^D) (%)
0-1	40.2	0.0146	0.9568	15.9	15.9	15.9
2-4	40.2	0.0088	1.3366	15.9	15.9	15.9
5-9	40.2	0.0081	1.3731	15.9	15.9	15.9
10-17	40.2	0.0081	1.3731	77.2	77.2	77.2
18-24	22.7	0.0088	1.3169	77.2	77.2	77.2
25-49	22.7	0.0042	1.6774	77.2	77.2	77.2
50-64	22.7	0.0043	1.7102	22.2	22.2	22.2
65+	28.8	0.0032	1.9374	22.2	22.2	22.2

Table 3. Vaccination Parameter Values for both the Mild and Severe Scenario.
Notes: [a]The Bass p_i and q_i values were fit when t is measured in months. See Section 3 for more details.

1.5 US Demographics and Contact matrix

The US population was modeled using the *US Census 2012 National Population Projections, Middle Series: Table 1. Projected Population by Single Year of Age, Sex, Race, and Hispanic Origin for the United States: 2012 to 2060* [26].

We used the 2013 projection, which lists the total US population as 316,438,601 persons ($\bar{N}$). θ_i is the probability of being in the ith age group.

Age Groups	0-1	2-4	5-9	10-14	15-17	18-19	20-24	25-29	30-34	35-39	40-44	45-49	50-54	55-59	60-64	65-69	70+
θ_i	0.027	0.038	0.065	0.065	0.039	0.027	0.072	0.068	0.067	0.062	0.066	0.067	0.071	0.067	0.057	0.046	0.095

Table 4. Distribution of Persons by Age in the US Population in 2013.

Like Medlock and Galvani [1], we parameterized the contact matrix based on a study of daily contacts of eight European countries [27]. We used the data collected for physical contacts only. There were 15 age groups in the European data, divided in five-year blocks, i.e., ages 0–4, 5–9,…, 65–69, and 70+.

We adjusted the 15 age groups to 17 age groups more useful to our study: **0-1, 2-4**, 5-9, 10-14, **15-17, 18-19**, 20-24, 25-29, 30-34, 35-39, 40-44, 45-49, 50-54, 55-59, 60-64, 65-69, and 70+. (Bold emphasis to illustrate changes.) We broke up the 0-4 year old and 15-19 year old age groups. The contact rates from the European study's age group 0–4 were assumed to apply equally to our age groups 0-1 and 2-4: $c_{1j} = c_{2j}$ and $c_{i1} = c_{i2}$, where subscripts 1 and 2 indicate age groups 1 and 2. Likewise, the contact rates from the European study's age group 15-19 were assumed to apply equally to our age groups 15-17 and 18-19.

The output of the contact matrix (c_{ij}) was a contact rate given in average contacts per day of age group *i* with age group *j*.

The final contact matrix is shown in Table 5.

	0-1	2-4	5-9	10-14	15-17	18-19	20-24	25-29	30-34	35-39	40-44	45-49	50-54	55-59	60-64	65-69	70+
**0-1**	32.43	32.43	15.61	5.23	2.98	2.98	4.57	8.74	13.45	11.08	8.06	4.77	4.61	4.64	5.09	3.97	2.93
**2-4**	32.43	32.43	15.61	5.23	2.98	2.98	4.57	8.74	13.45	11.08	8.06	4.77	4.61	4.64	5.09	3.97	2.93
**5-9**	15.61	15.61	79.62	13.75	4.22	4.22	2.94	6.14	10.20	13.01	10.03	4.85	4.63	4.00	3.91	3.98	3.02
**10-14**	5.23	5.23	13.75	96.96	15.20	15.20	3.16	2.04	4.74	8.60	11.38	6.86	3.91	2.30	2.47	2.57	3.67
**15-17**	2.98	2.98	4.22	15.20	86.62	86.62	13.88	4.19	2.76	4.19	8.08	8.99	4.71	2.76	1.47	1.55	2.62
**18-19**	2.98	2.98	4.22	15.20	86.62	86.62	13.88	4.19	2.76	4.19	8.08	8.99	4.71	2.76	1.47	1.55	2.62
**20-24**	4.57	4.57	2.94	3.16	13.88	13.88	34.35	15.09	7.30	4.70	5.40	6.76	5.96	3.78	2.18	1.92	2.06
**25-29**	8.74	8.74	6.14	2.04	4.19	4.19	15.09	21.46	11.18	6.30	4.84	5.10	6.53	5.20	3.86	2.45	2.17
**30-34**	13.45	13.45	10.20	4.74	2.76	2.76	7.30	11.18	15.66	11.44	6.85	5.47	4.84	5.16	5.28	3.72	2.09
**35-39**	11.08	11.08	13.01	8.60	4.19	4.19	4.70	6.30	11.44	15.86	8.97	5.54	4.43	3.91	5.08	4.42	2.72
**40-44**	8.06	8.06	10.03	11.38	8.08	8.08	5.40	4.84	6.85	8.97	13.43	8.94	5.76	3.86	4.70	4.69	3.45
**45-49**	4.77	4.77	4.85	6.86	8.99	8.99	6.76	5.10	5.47	5.54	8.94	11.03	7.35	4.82	3.56	3.27	4.16
**50-54**	4.61	4.61	4.63	3.91	4.71	4.71	5.96	6.53	4.84	4.43	5.76	7.35	11.02	8.01	4.62	3.68	4.15
**55-59**	4.64	4.64	4.00	2.30	2.76	2.76	3.78	5.20	5.16	3.91	3.86	4.82	8.01	12.55	7.16	5.53	3.39
**60-64**	5.09	5.09	3.91	2.47	1.47	1.47	2.18	3.86	5.28	5.08	4.70	3.56	4.62	7.16	11.90	8.67	5.41
**65-69**	3.97	3.97	3.98	2.57	1.55	1.55	1.92	2.45	3.72	4.42	4.69	3.27	3.68	5.53	8.67	11.95	8.20
**70+**	2.93	2.93	3.02	3.67	2.62	2.62	2.06	2.17	2.09	2.72	3.45	4.16	4.15	3.39	5.41	8.20	6.89

Table 5. Contact Matrix for Age-Structured Disease Transmission Model. Darker shading indicates more frequent contacts.

1.6 Initial Conditions

We required an initial estimate of the infected population at the time of the model start. Our model began August 1 in the year of the pandemic.

We began with an initial number of cases: C_0 at time t=0. We distributed these initial cases according to age based on the age distributions cited in the supplemental technical appendix in [18]. That is, we defined ϕ_k to be equal to the case probabilities by the kth age group shown in Table 6 below.

Age group	Case Probabilities (ϕ_k)
0–4	0.130
5–24	0.596
25–49	0.201
50–64	0.059
>65	0.014

Table 6. Distribution of Influenza Cases by Age.

In order to further distribute these cases among the 17 age groups in our model, we rescaled as follows:

We have already defined θ_i as the probability of an individual being in the ith age group where the age groups and their probabilities awere defined in Table 4.

Let y_k denote the membership of the kth age group where the age groups are defined as in Table 6 and k = 1,2,…,5.

$y_k = \sum \theta_i$ for all i age groups $\in$ k age groups

Probability that an influenza case is in the ith age group= $\dfrac{\theta_i}{y_k}$

$$I_i^U(t=0) = C_0\phi_k\left(\frac{\theta_i}{y_k}\right)$$

$$S_i^U(t=0) = \upsilon_i\left[(\theta_i)\overline{N} - I_i^U(t=0)\right]$$

$$S_i^R(t=0) = (1-\upsilon_i)\left[(\theta_i)\overline{N} - I_i^U(t=0)\right]$$

All other compartments were initially zero.

1.6.1 Mild Scenario

For simplicity, we adopted the ϕ_k values given in Table 6 despite the fact that they are based on data from the 2009-2010 pandemic. We set C_0 to be equal to 0.0001*($\bar{N}$) or ~32,000 persons. We primarily chose this value such that the peak of the pandemic influenza followed the peak of vaccination (see Figure 3 in the main text).

1.6.2 Severe Scenario

For simplicity, we adopted the ϕ_k values given in Table 6 despite the fact that they are based on data from the 2009-2010 pandemic. We set C_0 at 100 cases so that the peak of vaccination aligned with the peak of influenza.

1.6.3 Delayed Vaccination Campaign Start

We employed a delayed start to the vaccination campaign such that the vaccination became available at some time after August 1, when $t = t_V$, which we set to 30 days. At this time, we initiated the Bass diffusion process. Thus,

$$\omega_i(t < t_V) = 0$$

$$\omega_i(t = t_V) = p_i$$

2 H1N1 Face Validation of Age-Structured Disease Transmission Model

We performed a simple face validation of our age-structured disease transmission model using similar assumptions to those used by [28].

2.1 Epidemiologic Parameters

We set R_0 = 1.55 in the H1N1 Face Validation scenario.

Age Group	Incubation Time (τ_i^{-1}) (days) [7,9]	Duration of Infectiousness (γ_i^{-1}) (days) [10–14]	Influenza Attack Rate (π_i) (cases per 100 persons) [8,15,16]	[a]Per-Capita Hospitalization Rate (η_i) (hospitalizations per 100,000 persons) [8]	Per-Capita Death Rate (δ_i) (deaths per 100,000 persons) [8,17]
0-4	3	7	26.4	50.62*multiplier	0.39
5-17	3	7	26.4	19.74*multiplier	0.34
18-24	3	7	18.5	17.30*multiplier	0.48
25-49	3	7	18.5	13.86*multiplier	0.80
50-64	3	7	18.5	19.76*multiplier	1.38
65+	3	7	18.5	14.52*multiplier	0.87

Table 7. Epidemiological Parameter Values used to model the H1N1 Scenario.
[a]The per capita hospitalization rate is taken from the Aggregate Hospitalization and Deaths Reporting Activity (AHDRA), which was a special project initiated by the Centers for Disease Control and Prevention (CDC) during the pandemic. Because AHDRA numbers likely undercounted actual hospitalizations and deaths, the CDC developed a multiplier factor of 2.7 to account for underascertainment [18]. We also use the multiplier factor.

2.2 Vaccination Parameters

Age Group	Expected Vaccination Coverage (υ_i) (%) [24]	[a]Bass p_i	[a]Bass q_i	Vaccine Effectiveness against Influenza (ε_i) (%) [25]	Vaccine Effectiveness against Hospitalization (ε_i^H) (%)	Vaccine Effectiveness against Death (ε_i^D) (%)
0-1	41.2	0.0146	0.9569	15.9	15.9	15.9
2-4	41.2	0.0088	1.3366	15.9	15.9	15.9
5-9	30.6	0.0081	1.3731	15.9	15.9	15.9
10-17	30.6	0.0081	1.3731	77.2	77.2	77.2
18-24	18.5	0.0088	1.3169	77.2	77.2	77.2

25-49	18.5	0.0042	1.6774	77.2	77.2	77.2
50-64	18.5	0.0043	1.7102	22.2	22.2	22.2
65+	26.6	0.0032	1.9374	22.2	22.2	22.2

Table 8. Vaccination Parameter Values for the H1N1 Scenario.
Notes: [a]The Bass p_i and q_i values were fit when t is measured in months. See Section 3 for more details.

2.3 Demographics

The US population was modeled using the *US Census 2009 National Population Estimates for Resident Population: Estimates by Age, Sex, Race, and Hispanic Origin (Vintage 2009): April 1, 2000 to December 31, 2009* [29].

We used the August 2009 estimate, which lists the total US population as 307,251,662 persons.

Age Groups	0-1	2-4	5-9	10-14	15-17	18-19	20-24	25-29	30-34	35-39	40-44	45-49	50-54	55-59	60-64	65-69	70+
θ_i	0.028	0.042	0.067	0.065	0.041	0.029	0.070	0.071	0.065	0.067	0.068	0.074	0.071	0.062	0.052	0.038	0.091

Table 9. Distribution of Persons by Age in the US Population in August 2009.

2.4 Initial Conditions

We required an initial estimate of the infected population at the time of the model start. Our model began August 1 in the year of the pandemic. As before, we set an initial number of cases at time t=0, and distributed these initial cases according to age based on the age distributions cited in the supplemental technical appendix in [18].

Shrestha et al. provided national estimates for the number of H1N1 cases in the entire month of August [16]: 1,605,760 cases. Truly, we would have liked to know the number of infected persons on August 1. We set C_0 to be equal to 650,000 persons. We chose this value such that the peak of the pandemic influenza with a population that achieved vaccination coverage as described in Table 9 occurred around November 1.

We employed a delayed start to the vaccination campaign such that the vaccination became available at some time after August 1, when $t = t_V$, which we set to 60 days. This is consistent with the availability of H1N1 vaccine.

2.5 Results

Shrestha et al. provide national estimates of the number of cases, hospitalizations, and deaths for the 2009/2010 influenza period [16]. Our simulation covers the time period August 1, 2009-July 31, 2010. Therefore, we combine Shrestha et al.'s estimates for August 1-31, 2009 and September 1, 2009-April 10, 2010.

Output	Shrestha Estimate	H1N1 Calibration Run
Cases	57,784,980	129,523,253
Hospitalizations	260,539	314,897

Deaths	11,952	4,472

Table 10. Correspondence between Shrestha et al. burden estimates and H1N1 calibration run of the influenza transmission model.

With respect to influenza cases, we calculate a little over double what Shrestha et al. estimate in the H1N1 scenario. However, considering the very different methods used to arrive at this figure, we are happy to be this close. We were heartened to be so close on hospitalizations, which is the measure that CDC deems to be most reliable. On deaths, we are using the AHDRA reporting figures, which is an underascertainment, but CDC did not generate a multiplier to be applied to the AHDRA death totals as it did for hospitalizations [18]. Shrestha et al. apply a multiplier to the their hospitalizations figure [16]. If we do the same to calculate deaths, then we calculate 13,273 deaths, which is unsurprising given the correspondence among hospitalizations.

3 Vaccine Safety Surveillance System Model

We simulated sequential database surveillance for a) influenza vaccination-associated febrile seizures and b) influenza vaccination-associated Guillain-Barré Syndrome (GBS). We simulated surveillance by modeling an "enhanced" Post-licensure Rapid Immunization Safety Monitoring (PRISM) system, which is the supporting infrastructure for seasonal influenza surveillance [23]. Broadly speaking, we performed the following steps:

1 - Calculated database-specific chronological exposure estimates for each database using an age-structured delay differential equation model;
2 – Adjusted the chronological exposure estimates to reflect database-specific processing delays;
3 – Used the adjusted exposure estimates, along with other adjustments, to generate information on outcomes of interest;
4 - Aggregated database-specific information and performed sequential statistical analyses on simulated data.

A more detailed discussion of the methodology of these simulations is discussed elsewhere [30]. All analyses were completed using MATLAB® and R.

3.1 Bass Adoption Model for Influenza Vaccine

We used an age-structured delay differential equation model to describe the adoption of influenza vaccine within the surveillance population. Figure 1 shows the flow of persons.

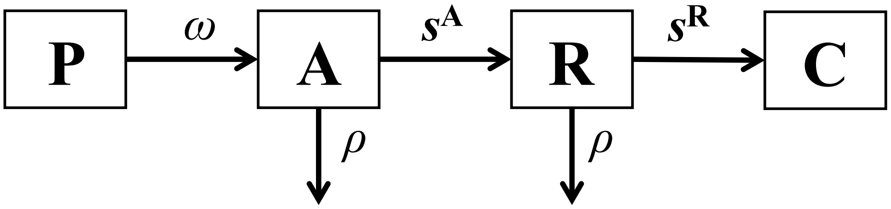

Figure 2. Age-Structured Delay Differential Equation Model of the flow of persons.

3.1.1 Model Variables

$P_i^k(t)$: the number of potential adopters in the *k*th database in the *i*th age group at time *t*;

$A_i^k(t)$: the number of adopters who have not yet entered the risk window in the *k*th database in the *i*th age group at time *t*;

$R_i^k(t)$: the number adopters in the risk window ("at risk") the *k*th database in the *i*th age group at time *t*;

$C_i^k(t)$: the number of adopters who have exited the risk window in the *k*th database in the *i*th age group at time *t*;

$V_i^k(t)$: the number of total adopters in the *k*th database in the *i*th age group at time *t*;

$E_i^k(t)$: the cumulative amount of exposed-person time in the *k*th database in the *i*th age group at time *t*;

$\omega_i^k(t)$: the conditional probability that an individual in age group *i* in database *k* will vaccinate at exactly time *t* since introduction of the vaccination, given that the individual has not vaccinated before that time; also known as the hazard function;

δ_1 : the time period after a person has been exposed to a medical product but before the person is "at risk" for experiencing the outcome of interest, also known as the induction period or the latent period.

δ_2 : the time period when a person is "at risk" of experiencing an outcome of interest following some exposure of interest, also know as the risk window.

ρ_i : the incidence rate of the outcome expected under the null hypothesis (i.e., that there is no excess risk in the treatment group).

3.1.2 Model Equations

The model is a system of 7×*n*×*m* ordinary differential equations, with *n* age-groups from *i*=1,2,…,*n, and m* databases from *k*= *i*=1,2,…,*m*. The time unit *t* is in months. The model equations are as follows:

$$\frac{dP_i^k}{dt} = -\omega_i^k(t)P_i^k(t) \tag{S2-1}$$

$$\frac{dA_i^k}{dt} = \omega_i^k(t)P_i^k(t) - \rho_i A_i^k(t) - s_i^A\left[\omega_i^k(t-\delta_1)P_i^k(t-\delta_1)\right] \tag{S2-2}$$

$$\frac{dR_i^k}{dt} = s_i^A\left[\omega_i^k(t-\delta_1)P_i^k(t-\delta_1)\right] - \rho_i R_i^k(t) - s_i^R s_i^A\left[\omega_i^k(t-\delta_1-\delta_2)P_i^k(t-\delta_1-\delta_2)\right] \tag{S2-3}$$

$$\frac{dC_i^k}{dt} = s_i^R s_i^A\left[\omega_i^k(t-\delta_1-\delta_2)P_i^k(t-\delta_1-\delta_2)\right] \tag{S2-4}$$

$$\frac{dE_i^k}{dt} = R_i^k(t) \tag{S2-5}$$

$$\frac{dV_i^k}{dt} = \frac{dA_i^k}{dt} + \frac{dR_i^k}{dt} + \frac{dC_i^k}{dt} \tag{S2-6}$$

$$\frac{d\omega_i^k}{dt} = \frac{q_i^k}{\Omega^k}\left(\frac{dV_i^k}{dt}\right) \tag{S2-7}$$

$$s_i^A = e^{-\rho_i \delta_1} \tag{2-8}$$

$$s_i^R = e^{-\rho_i \delta_2} \tag{2-9}$$

where s_i^A and s_i^R are survivability constants that result from censoring individuals who experience the outcome of interest. The occurrence of the outcome of interest was modeled as a Poisson process.

3.1.3 Vaccination Adoption Function

The influenza vaccination adoption function is modeled as a Bass diffusion process [2] given by the following equation:

$$\omega_i^k(t) = p_i^k + \frac{q_i^k}{\Omega^k} V_i^k(t) \tag{2-10}$$

where p_i^k and q_i^k are the Bass coefficients of innovation and imitation, respectively. Ω^k is the total population of those likely to vaccinate.

3.1.4 Initial Conditions for Delay Differential Equation Model

$$P_i^k(t=0) = \Omega^k$$
$$\omega_i^k(t=0) = p_i^k$$

All other compartments are zero at time t=0.

3.2 Calculating Database Lag

For each database k, it was necessary to delay the chronological exposure by a processing time, allowing for “claims” to be processed [31]. This time is a random variable estimated by empirical data.

Let $E_i^k(t)$ be a vector of <u>cumulative</u> exposure (in person-time) for the ith age group in the kth database and $e_i^k = E_i^k(y) - E_i^k(y-1)$ for y=1,2,…,w where w is the number of discrete increments in $E_i^k(t)$

Let $F_\theta(t)$ be the cumulative distribution function of the processing time delay θ and $p_\theta(z) = F_\theta(z) - F_\theta(z-1)$ for z=1,2,…,x where x is the number of discrete increments in $F_\theta(t)$.

It is important to ensure that t is in consistent units in the processing time delay and in the exposure vectors above. If that is not the case, both vectors need to be converted to a common unit.

Let M be a temporary matrix of exposures redistributed to account for the processing delay times where

$$\mathrm{M} = e_i^k\left[\left(p_\theta\right)^{\mathrm{T}}\right] \tag{2-11}$$

M will be size (w,x). M is then horizontally concatenated to a matrix of zeros of size (w, w-1). Now, each row of this new matrix, M hat, is then shifted right as in the example below to account for delays. In the example, w=4, x=2.

$$\mathrm{M} = \begin{bmatrix} a & b \\ c & d \\ e & f \\ g & h \end{bmatrix} \quad \begin{bmatrix} 0 & 0 & 0 \\ 0 & 0 & 0 \\ 0 & 0 & 0 \\ 0 & 0 & 0 \end{bmatrix} \quad \hat{\mathrm{M}} = \begin{bmatrix} a & b & 0 & 0 & 0 \\ 0 & c & d & 0 & 0 \\ 0 & 0 & e & f & 0 \\ 0 & 0 & 0 & g & h \end{bmatrix}$$

$\hat{e}_i^k = \hat{\mathrm{M}}^{\mathrm{T}} 1^{\mathrm{T}}$ where 1=(1,1,...,1) is a vector of length w.

From $\hat{e}_i^k$, we can calculate the adjusted vector of cumulative exposure $\hat{E}_i^k$ which has length w+x-1.

3.3 Generate Outcomes of Interest

3.3.1 Variable Definition

$\mathrm{Z}_i^k(t)$: the number of true positive cases among the treatment population identified in the kth database for the ith age group at time t;

$\Upsilon_i^k(t)$: the number of false positive cases among the treatment population identified in the kth database for the ith age group at time t;

$\mathrm{B}_i^k(t)$: the number of false negative cases among the treatment population identified in the kth database for the ith age group at time t;

$T_i^k(t)$: the number of true positive cases among the comparator population identified in the *k*th database for the *i*th age group at time *t*;

$X_i^k(t)$: the number of false positive cases among the comparator population identified in the *k*th database for the *i*th age group at time *t*;

$Y_i^k(t)$: the number of false negative cases among the comparator population identified in the *k*th database for the *i*th age group at time *t*;

$\hat{\psi}_i^k$: the **effective** positive predictive value among the treatment population in the kth database for the ith age group

ψ_i^k: the positive predictive value among the comparator population in the kth database for the ith age group

ϕ_i^k: the sensitivity of the algorithm used to detect the outcome of interest in the *k*th database for the *i*th age group

r: the risk of the outcome of interest given as a ratio

ρ_i: the incidence rate of the outcome expected under the null hypothesis (i.e., that there is no excess risk in the treatment population), also known as the background rate among the comparator population

3.3.2 Outcomes in the Treatment Population

Using the data on exposure, we generated 10,000 simulations of outcome patterns for analysis according to the following equations. That is, we repeatedly used a Poisson random number generator for variables distributed as Poisson.

$$Z_i^k(t) = \hat{e}_i^k(t)\left[\text{Pois}\left(\phi_i^k r \rho_i\right)\right] \tag{2-12}$$

$$\Upsilon_i^k(t) = \hat{e}_i^k(t)\left[\text{Pois}\left(\left[\frac{1}{\hat{\psi}_i^k} - 1\right]\phi_i^k r \rho_i\right)\right] \tag{2-13}$$

$$B_i^k(t) = \hat{e}_i^k(t)\left[\text{Pois}\left(\left[1 - \phi_i^k\right] r \rho_i\right)\right] \tag{2-14}$$

where $\hat{\psi}_i^k = \dfrac{\psi_i^k r(1-\rho_i)}{r(\psi_i^k - \rho_i) + 1 - \psi_i^k}$ was calculated as described in [32,33]. (2-15)

3.3.3 Outcomes in the Comparator Population

We also generated outcomes under the null hypothesis, but these outcomes were deterministic and do not change throughout the 10,000 simulations:

$$T_i^k(t) = \hat{e}_i^k(t)\left(\phi_i^k r \rho_i\right) \tag{2-16}$$

$$X_i^k(t) = \hat{e}_i^k(t)\left(\left[\frac{1}{\psi_i^k} - 1\right]\phi_i^k r \rho_i\right) \tag{2-17}$$

$$Y_i^k(t) = \hat{e}_i^k(t)\left(\left[1 - \phi_i^k\right] r \rho_i\right) \tag{2-18}$$

3.4 Sequential Statistical Analyses

Given the simulated data on exposure and outcomes of interest, we applied the Poisson Maximized Sequential Probability Ratio Test (MaxSPRT) [34] to the simulated data, which was part of the current analytical plan when monitoring seasonal influenza vaccination [35]. Briefly, the Poisson MaxSPRT compared current adopters of influenza vaccination to historical adopters of influenza vaccination to detect an elevated risk of designated adverse events (i.e., outcomes of interest). The null hypothesis was that the risk after influenza vaccination in the pandemic period is no greater than the risk after influenza vaccination in past seasons.

The null hypothesis of no increased risk will be rejected if the test statistic, the log likelihood ratio (LLR), reaches an upper boundary, known as the critical value. The null hypothesis will not be rejected if the total number of cases of the designated adverse event surpasses a pre-specified "upper limit" for surveillance. Alternatively, it was possible to fail to reach either boundary condition because of a lack of cases, which ultimately implies a failure to reject the null hypothesis.

The LLR was calculated as follows:

$$LLR = \mu_t - c_t + c_t \ln\left(\frac{c_t}{\mu_t}\right) \tag{2-19}$$

where c_t is the number of observed adverse events (cases) in time period *t*; μ_t is the number of expected events (cases) under the null hypothesis in time period t as given by

ρ_i . It is only calculated when $c_t \geq \mu_t$ and is 0 otherwise.

For each outcome of interest, the critical value of the LLR was calculated by the user-specified upper limit of expected events and alpha level (i.e., Type I error). Upper limits in these analyses are historically selected based on the approximate number of events that would be expected under the null hypothesis in the risk interval. This value is incremented up to prevent reaching an end of surveillance before the surveillance period is over. One-tailed tests were used for alpha (i.e., Type I error).

3.5 Parameters

All surveillance parameters were modeled on seasonal influenza surveillance planned for the 2013-2014 season [35].

3.5.1 Febrile Seizures

3.5.1.1 Surveillance Parameters

The comparator outcome rate was based on data on individuals in the PRISM system that had previously received an inactivated trivalent influenza vaccine and experienced a febrile seizure in the risk window. All other epidemiologic parameters are based on the protocol or discussion with PRISM epidemiologists.

**Parameter**		**Value**
Design		Cohort Design with Poisson MaxSPRT
Upper Limit		25 events
Comparator Outcome Rate (ρ_i)	0-1	138 events/4,289,583 days at risk
	2-4	35 events /3,201,643 days at risk
Induction Period (δ_1)		0 days
Risk Window (δ_2)		2 days (0-1.9999 days)
Type I Error (α)		0.05
Effect Size of Interest (*r*)		Incidence Rate Difference of ~150 excess febrile seizures per 100,000 doses

Table 11. Surveillance Parameters for Influenza Vaccination-Febrile Seizures Surveillance.

3.5.1.2 Database Parameters

The size of the surveillance population was determined by averaging adopters of influenza vaccination in the PRISM system over three previous influenza seasons (2008-2009, 2009-2010, 2010-2011). The processing delay was an empirical distribution function based on historical claims originating from the Emergency Department.

Parameters		Values	References
Database Size	0-1	502,641	NA
	2-4	314,322	NA
Positive Predictive Value (ψ_i^k)		0.87	[36]
Sensitivity (ϕ)		0.80	[36]
[a]Bass *p*	0-1	0.0146	NA
	2-4	0.0088	NA
[a]Bass *q*	0-1	0.9568	NA
	2-4	1.3366	NA

Table 12. Database Parameters for Influenza Vaccination-Febrile Seizures Surveillance.
[a]Bass *p* and Bass *q* are listed by age group, but they are also calculated by database. We are not permitted to share database-specific information and so a PRISM-wide coefficient is shown. Bass *p* and Bass *q* were fit when *t* is measured in months.

3.5.2 Guillain-Barré Syndrome

3.5.2.1 Surveillance Parameters

The comparator outcome rate was based on data on individuals in the PRISM system that had previously received an inactivated trivalent influenza vaccine and experienced a febrile seizure in the risk window. All other epidemiologic parameters were based on the protocol or discussion with PRISM epidemiologists.

Parameter		Value
Design		Cohort Design with Poisson MaxSPRT
Upper Limit		30 events
Comparator Outcome Rate (ρ_i)	0-1	1.08 events/ 100,000 person-years
	2-4	1.58 events/ 100,000 person-years
	5-17	2.73 events/ 100,000 person-years
	18-24	2.57 events/ 100,000 person-years

	25-49	5.10 events/ 100,000 person-years
	50-64	5.25 events/ 100,000 person-years
	65+	5.37 events/ 100,000 person-years
Induction Period (δ_1)		1 day (0-0.9999 days)
Risk Window (δ_2)		42 days (1-42.9999 days)
Type I Error (α)		0.05
Effect Size of Interest (*r*)		Incidence Rate Difference of 40 excess GBS cases per million doses

Table 13. Surveillance Parameters for Influenza Vaccination-GBS Surveillance.

3.5.2.2 Database Parameters

The size of the surveillance population was determined by averaging adopters of influenza vaccination in the PRISM system over two previous influenza seasons (2009-2010, 2010-2011). The processing delay was an empirical distribution function based on historical claims originating from inpatient services at the hospital.

**Parameters**		**Values**	**References**
Database Size	0-1	528,780	NA
	2-4	304,989	NA
	5-17	694,638	NA
	18-24	112,035	NA
	25-49	875,290	NA
	50-64	985,617	NA
	65+	814,131	NA
[a]Bass *p*	0-1	0.0146	NA
	2-4	0.0088	NA
	5-17	0.0081	NA
	18-24	0.0088	NA
	25-49	0.0042	NA
	50-64	0.0043	NA
	65+	0.0032	NA
[a]Bass *q*	0-1	0.9569	NA

	2-4	1.3366	NA
	5-17	1.3731	NA
	18-24	1.3169	NA
	25-49	1.6774	NA
	50-64	1.7102	NA
	65+	1.9374	NA
Positive Predictive Value (ψ_i^k)		0.55	[37]
Sensitivity (ϕ)		1.0	[37]

Table 14. Database Parameters for Influenza Vaccination-GBS Surveillance.
[a]Bass p and Bass q are listed by age group, but they are also calculated by database. We are not permitted to share database-specific information and so a PRISM-wide coefficient is shown. Bass p and Bass q were fit when t is measured in months.

4 Additive Multi-Attribute Utility Function

In multi-criteria decision analyses (MCDAs), outcomes of decisions are characterized by multiple, often competing criteria or attributes, some of which may be less important than others but nevertheless important enough to be considered in the analysis. Outcomes are thus multivariate. Decision makers must consider all criteria when evaluating possible decision options. A multi-attribute utility (MAU) function is a mathematical function used to characterize the overall value (or "utility") of each outcome relative to the others to represent the judgment of the decision maker [38]. The MAU is used to weight and combine multiple attributes into a single figure of merit whose expected value was maximized to select the preferred regulatory decision.

Using an expert panel preference solicitation process, we developed an additive MAU function. We also considered multiplicative and multi-linear formulations but ruled these out in favor of the simplicity of the additive model.

We use the following functional form:

$$U(x_1,\ldots,x_m) = \sum_{i=1}^{m} k_i U_i(x_i) \qquad \sum_{i=1}^{m} k_i = 1$$

where x_i are the attributes of interest and k_i are the weights associated with those attributes.

We evaluated four attributes as follows:

Attribute	Measure	Range
1. Expected vaccination benefit from averted influenza	Composite index of influenza-associated cases averted, hospitalizations averted, and deaths averted	Range was re-calculated based on low and high values under a particular "run" of the influenza transmission model.
2. Expected vaccination risk from influenza vaccine-associated febrile seizures	Attributable cases of febrile seizures	Range was re-calculated based on low and high values under a particular "run" of the influenza transmission model.
3. Expected vaccination risk from influenza vaccine-associated Guillain-Barré Syndrome (GBS)	Attributable cases of GBS	Range was re-calculated based on low and high values under a particular "run" of the influenza transmission model.
4. Expected change in public vaccination-seeking behavior in future seasons as a direct consequence of public reaction to changes in federal vaccination policy during the pandemic.	Qualitative Variable	1. No Change<br>2. Minor Change (Anticipated 10% reduction in vaccination-seeking behavior in future seasons)<br>3. Major Change (Anticipated 10% reduction in vaccination-seeking behavior in future seasons)

Table 15. Attributes used in Multi-Attribute Utility Function

Every outcome of the decision model is represented as a vector of values for each of these four attributes. One example outcome follows:

Attribute 1: 23,940,000 saved infections, 18,000 saved hospitalizations, 9,794 saved deaths
Attribute 2: 6700 excess febrile seizures
Attribute 3: 0 excess GBS cases
Attribute 4: Minor Change, 10% reduction in vaccine-seeking behavior

4.1 Expert Panel Preference Elicitation

We convened an expert panel of six physicians who were currently serving or had previously served on vaccination-related federal advisory boards. All physicians were pediatricians. They were asked as individuals to assume the role of regulatory decision makers, i.e., their values served as proxy for decision makers may have to actually make such decisions.

We used a fractional factorial design to select sets of outcomes (i.e., combinations of levels of the attributes listed above) for evaluation; each panelist was asked to consider each outcome as a whole and to rank the set of outcomes, then to assign the best outcome (in that panelist's assessment) the value of 100 and the worst outcome a value of 0, then to rate the remaining outcomes relative to the anchor points. This process and facilitated discussion of ratings among the panelists was to help them think deeply about how each outcome attribute weighed in the decision process and what tradeoffs were made in thinking about overall attractiveness or unattractiveness of an outcome. Later in the day, using a series of steps [38–40] each panelist was led through a process of developing the disaggregated parts of a MAU function which could be used to assign values to outcomes.

We performed separate elicitations conditional on the mild scenario and on the severe scenario, thereby creating separate MAU functions for the two situations. We combined each expert panelist's MAU function to derive the "average" federal decision-maker.

Attribute	Expected Vaccination Benefit	Expected Vaccine-associated risk from Febrile Seizures	Expected Vaccine-associated risk from GBS	Expected Future Change in Vaccine-Seeking Behavior
Mild Scenario[a]	0.55	0.01	0.16	0.28
Severe Scenario[b]	0.664	0.012	0.074	0.250

Table 16. Averaged Scaling Constants of the Expert Panel for Multi-Criteria Decision Analysis.
Notes: A scaling constant represents the relative weight given to each criterion in the utility function and together, they must sum to 1.
[a]The mild scenario was characterized by low transmissibility, low severity, and the peak of vaccination preceded the peak of influenza transmission.

[b]The severe scenario was characterized by high transmissibility, moderate-to-high severity, and the peak of vaccination occurred concurrently with the peak of influenza transmission.

Following the initial day-long expert panel preference elicitation process, we re-contacted each expert with an analysis of the correlation between their user-valued ratings and the model of their individual preferences to assess whether we had accurately captured such preferences. We provided each expert the opportunity to change or iterate on his or her individualized model. Most experts retained their original preferences.

4.1.1 Mild Scenario Permutation

Figure 3 shows the correspondence between the user-valued ratings and the derived model in the set of 31 mild scenario permutations.

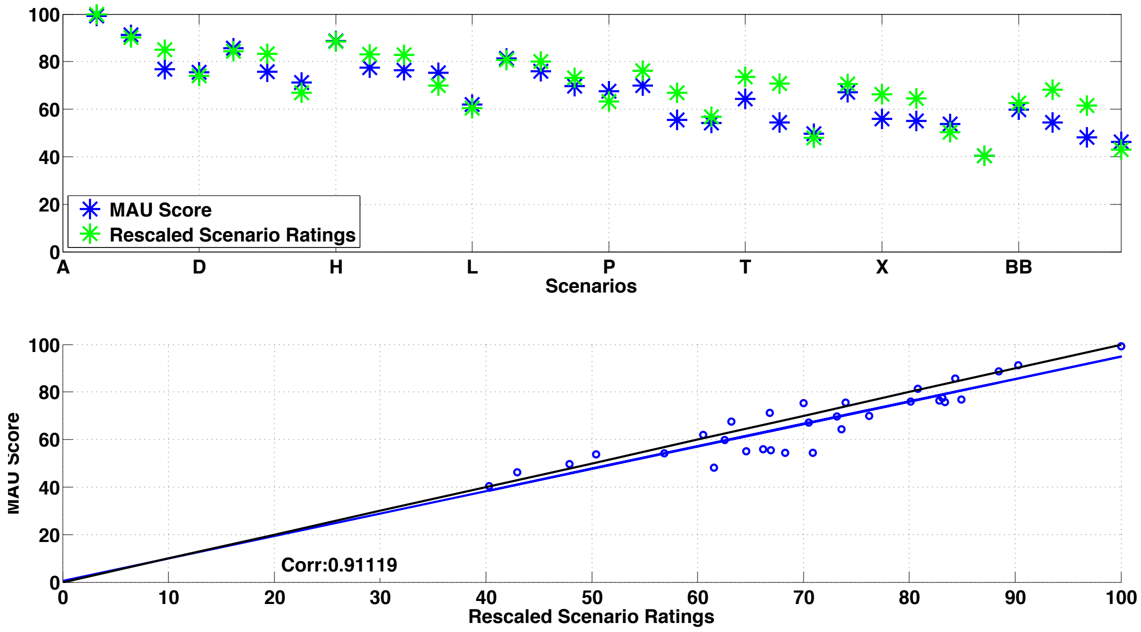

Figure 3. Correlation between Multi-Attribute Utility Function model and Scenario Rating for the "Average" decision-maker for the Mild Scenario. Abbreviations: MAU, multi-attribute utility

The upper panel shows the utility computed by the MAU in blue compared to the utility specified by the expert panel (green). In the lower panel, the black line is perfect correlation between the model and expert panel ratings. The actual correlation coefficient of the model and the expert panel ratings is displayed, and the blue line is the regression.

4.1.2 Severe Scenario

Figure 4 shows the correspondence between the user-valued ratings and the derived model in the set of 31 severe scenario permutations.

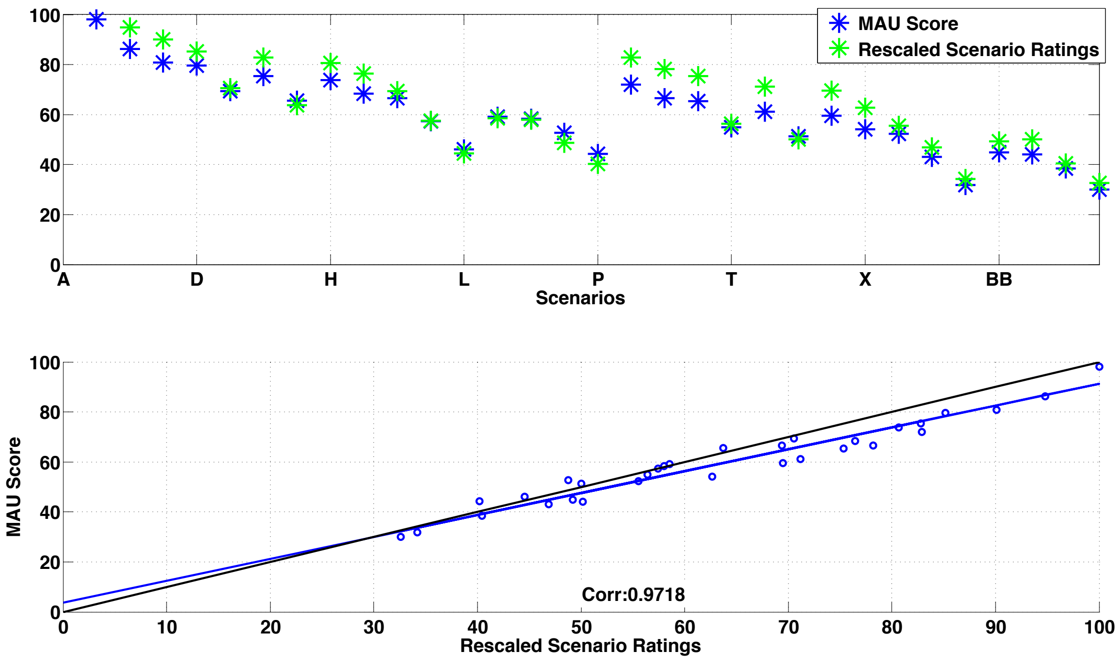

Figure 4. Correlation between Multi-Attribute Utility Function model and Scenario Rating for the "Average" decision-maker for the Severe Scenario. Abbreviations: MAU, multi-attribute utility

The upper panel shows the utility computed by the MAU in blue compared to the utility specified by the expert panel (green). In the lower panel, the black line is perfect correlation between the model and expert panel ratings. The actual correlation coefficient of the model and the expert panel ratings is displayed, and the blue line is the regression.

4.2 Conditional Probability Elicitation

Our regulatory decision model is concerned with the general public's reaction to federal vaccination policy changes and how that reaction translates into future vaccination seeking behavior. We lacked any public preference surveys similar to those described in [41] regarding changes in vaccination-seeking behavior as a result of government policy and therefore, we decided to elicit conditional predictions of the likelihoods of various reactions from the expert panel.

For each of the four decision options examined in our regulatory decision model, we asked the question:

"We are experiencing a {Mild/Severe} pandemic and an information signal of vaccine-associated risk is received by federal decision-makers, and they select {Decision Option 1-4}, then what is the probability that:
a. Public does not change their future vaccination-seeking behavior.
b. Public responds by reducing their future vaccination-seeking behavior by 10%.
c. Public responds by reducing their future vaccination-seeking behavior by 25%."

A visual representation of this question is shown in Figure 5.

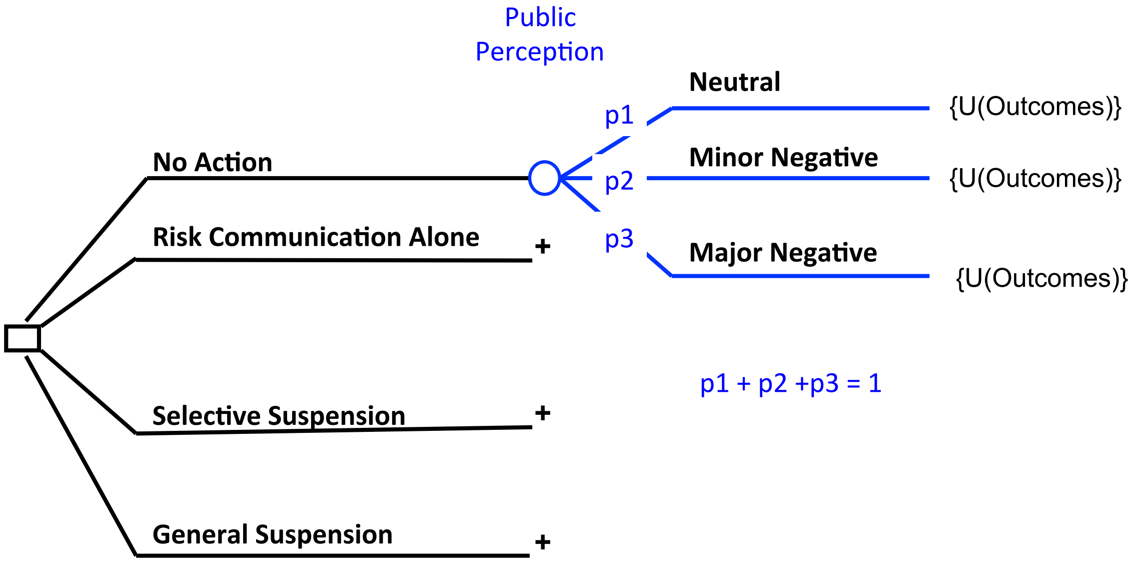

Figure 5. Diagram of Conditional Probability Elicitation

4.2.1 Mild Scenario Conditional Probabilities

	Reduction in Vaccine Seeking Behavior		
	No Change (0%)	Minor Change (-10%)	Major Change (-25%)
	Mild Scenario		
No Action	0.483	0.133	0.383
Risk Communication Alone	0.473	0.227	0.300
Selective Suspension	0.410	0.353	0.237
General Suspension	0.350	0.183	0.467

Table 17. Anticipated Changes in Vaccine-Seeking Behavior in Future Seasons associated with Four Regulatory Responses.

Notes: This is the averaged probability elicited from the expert panel. If a particular regulatory response is selected in the model, then each row represents the probability of the three levels (i.e., note that each row sums to 1.0). Therefore, regulatory responses with the highest probability of no change are associated with the highest levels of future vaccine-seeking behavior.

4.2.2 Severe Scenario

	Reduction in Vaccine Seeking Behavior		
	No Change (0%)	Minor Change (-10%)	Major Change (-25%)
	Severe Scenario		
No Action	0.533	0.083	0.383
Risk Communication Alone	0.590	0.190	0.220

Selective Suspension	0.517	0.257	0.227
General Suspension	0.573	0.243	0.183

Table 18. Anticipated Changes in Vaccine-Seeking Behavior in Future Seasons associated with Four Regulatory Responses.

Notes: This is the averaged probability elicited from the expert panel. If a particular regulatory response is selected in the model, then each row represents the probability of the three levels (i.e., note that each row sums to 1.0). Therefore, regulatory responses with the highest probability of no change are associated with the highest levels of future vaccine-seeking behavior.

5 REFERENCES

1. Medlock J, Galvani AP (2009) Optimizing influenza vaccine distribution. Science 325: 1705–1708. doi:10.1126/science.1175570.

2. Bass FM (1969) A New Product Growth for Model Consumer Durables. Management Science 15: 215–227.

3. Jewell NP, Lei X, Ghani AC, Donnelly CA, Leung GM, et al. (2007) Non-parametric estimation of the case fatality ratio with competing risks data: an application to Severe Acute Respiratory Syndrome (SARS). Stat Med 26: 1982–1998. doi:10.1002/sim.2691.

4. van den Driessche P, Watmough J (2002) Reproduction numbers and sub-threshold endemic equilibria for compartmental models of disease transmission. Math Biosci 180: 29–48.

5. Diekmann O, Heesterbeek JA, Metz JA (1990) On the definition and the computation of the basic reproduction ratio R0 in models for infectious diseases in heterogeneous populations. J Math Biol 28: 365–382.

6. Reed C, Biggerstaff M, Finelli L, Koonin LM, Beauvais D, et al. (2013) Novel framework for assessing epidemiologic effects of influenza epidemics and pandemics. Emerging Infect Dis 19: 85–91. doi:10.3201/eid1901.120124.

7. Boëlle P-Y, Ansart S, Cori A, Valleron A-J (2011) Transmission parameters of the A/H1N1 (2009) influenza virus pandemic: a review. Influenza Other Respi Viruses 5: 306–316. doi:10.1111/j.1750-2659.2011.00234.x.

8. Jhung MA, Swerdlow D, Olsen SJ, Jernigan D, Biggerstaff M, et al. (2011) Epidemiology of 2009 pandemic influenza A (H1N1) in the United States. Clin Infect Dis 52 Suppl 1: S13–26. doi:10.1093/cid/ciq008.

9. Donnelly CA, Finelli L, Cauchemez S, Olsen SJ, Doshi S, et al. (2011) Serial intervals and the temporal distribution of secondary infections within households of 2009 pandemic influenza A (H1N1): implications for influenza control recommendations. Clin Infect Dis 52 Suppl 1: S123–130. doi:10.1093/cid/ciq028.

10. Bhattarai A, Villanueva J, Palekar RS, Fagan R, Sessions W, et al. (2011) Viral shedding duration of pandemic influenza A H1N1 virus during an elementary school outbreak--Pennsylvania, May-June 2009. Clin Infect Dis 52 Suppl 1: S102–108. doi:10.1093/cid/ciq026.

11. Esposito S, Daleno C, Baldanti F, Scala A, Campanini G, et al. (2011) Viral shedding in children infected by pandemic A/H1N1/2009 influenza virus. Virol J 8: 349. doi:10.1186/1743-422X-8-349.

12. Suess T, Buchholz U, Dupke S, Grunow R, an der Heiden M, et al. (2010) Shedding and transmission of novel influenza virus A/H1N1 infection in households--Germany, 2009. Am J Epidemiol 171: 1157–1164. doi:10.1093/aje/kwq071.

13. Kay M, Zerr DM, Englund JA, Cadwell BL, Kuypers J, et al. (2011) Shedding of pandemic (H1N1) 2009 virus among health care personnel, Seattle, Washington, USA. Emerging Infect Dis 17: 639–644. doi:10.3201/eid1704.100866.

14. Tuite AR, Greer AL, Whelan M, Winter A-L, Lee B, et al. (2010) Estimated epidemiologic parameters and morbidity associated with pandemic H1N1 influenza. CMAJ 182: 131–136. doi:10.1503/cmaj.091807.

15. Dawood FS, Iuliano AD, Reed C, Meltzer MI, Shay DK, et al. (2012) Estimated global mortality associated with the first 12 months of 2009 pandemic influenza A H1N1 virus circulation: a modelling study. Lancet Infect Dis 12: 687–695. doi:10.1016/S1473-3099(12)70121-4.

16. Shrestha SS, Swerdlow DL, Borse RH, Prabhu VS, Finelli L, et al. (2011) Estimating the burden of 2009 pandemic influenza A (H1N1) in the United States (April 2009-April 2010). Clin Infect Dis 52 Suppl 1: S75–82. doi:10.1093/cid/ciq012.

17. Louie JK, Jean C, Acosta M, Samuel MC, Matyas BT, et al. (2011) A review of adult mortality due to 2009 pandemic (H1N1) influenza A in California. PLoS ONE 6: e18221. doi:10.1371/journal.pone.0018221.

18. Reed C, Angulo FJ, Swerdlow DL, Lipsitch M, Meltzer MI, et al. (2009) Estimates of the prevalence of pandemic (H1N1) 2009, United States, April-July 2009. Emerging Infect Dis 15: 2004–2007. doi:10.3201/eid1512.091413.

19. Longini IM Jr, Halloran ME, Nizam A, Yang Y (2004) Containing pandemic influenza with antiviral agents. Am J Epidemiol 159: 623–633.

20. Glezen WP (1996) Emerging infections: pandemic influenza. Epidemiol Rev 18: 64–76.

21. Glezen WP (1993) Influenza surveillance in an urban area. Can J Infect Dis 4: 272–274.

22. Serfling RE, Sherman IL, Houseworth WJ (1967) Excess pneumonia-influenza mortality by age and sex in three major influenza A2 epidemics, United States, 1957-58, 1960 and 1963. Am J Epidemiol 86: 433–441.

23. Nguyen M, Ball R, Midthun K, Lieu TA (2012) The Food and Drug Administration's Post-Licensure Rapid Immunization Safety Monitoring program: strengthening the federal vaccine safety enterprise. PharmacoepidemiolDrug Saf 21 Suppl 1: 291–297. doi:10.1002/pds.2323.

24. Centers for Disease Control and Prevention (CDC) (2011) Final estimates for 2009–10 Seasonal Influenza and Influenza A (H1N1) 2009 Monovalent Vaccination Coverage – United States, August 2009 through May, 2010. Available: http://www.cdc.gov/flu/professionals/vaccination/coverage_0910estimates.htm. Accessed 16 August 2013.

25. Griffin MR, Monto AS, Belongia EA, Treanor JJ, Chen Q, et al. (2011) Effectiveness of non-adjuvanted pandemic influenza A vaccines for preventing pandemic influenza acute respiratory illness visits in 4 U.S. communities. PLoS ONE 6: e23085. doi:10.1371/journal.pone.0023085.

26. U. S. Census Bureau (2012) 2012 National Population Projections: Downloadable Files. Available: http://www.census.gov/population/projections/data/national/2012/downloadablefiles.html. Accessed 16 August 2013.

27. Mossong J, Hens N, Jit M, Beutels P, Auranen K, et al. (2008) Social contacts and mixing patterns relevant to the spread of infectious diseases. PLoS Med 5: e74. doi:10.1371/journal.pmed.0050074.

28. Kostova D, Reed C, Finelli L, Cheng P-Y, Gargiullo PM, et al. (2013) Influenza Illness and Hospitalizations Averted by Influenza Vaccination in the United States, 2005-2011. PLoS ONE 8: e66312. doi:10.1371/journal.pone.0066312.

29. US Census Bureau DID (2009) Population Estimates. Available: http://www.census.gov/popest/data/national/asrh/2009/2009-nat-detail.html. Accessed 27 June 2014.

30. Maro JC, Brown JS, Kulldorff M (2013) Medical product safety surveillance: how many databases to use? Epidemiology 24: 692–699. doi:10.1097/EDE.0b013e31829dde59.

31. Greene SK, Kulldorff M, Yin R, Yih WK, Lieu TA, et al. (2011) Near real-time vaccine safety surveillance with partially accrued data. PharmacoepidemiolDrug Saf 20: 583–590. doi:10.1002/pds.2133.

32. Green MS (1983) Use of predictive value to adjust relative risk estimates biased by misclassification of outcome status. AmJEpidemiol 117: 98–105.

33. Brenner H, Gefeller O (1993) Use of the positive predictive value to correct for disease misclassification in epidemiologic studies. AmJEpidemiol 138: 1007–1015.

34. Kulldorff M, Davis RL, Kolczak M, Lewis E, Lieu TA, et al. (2011) A maximized sequential probability ratio test for drug and vaccine safety surveillance. Seq Anal 30: 58–78. doi:10.1080/07474946.2011.539924.

35. Yih WK, Sandhu S, Nguyen M, Zichittella L, McMahill-Walraven CN, et al. (2013) Accessing the Freshest Feasible Data for Conducting Active Influenza Vaccine

Safety Surveillance (PRISM). Available: http://www.mini-sentinel.org/work_products/PRISM/Mini-Sentinel_PRISM_Active-Influenza-Vaccine-Safety-Surveillance-Protocol.pdf. Accessed 19 August 2013.

36. Shui IM, Shi P, Dutta-Linn MM, Weintraub ES, Hambidge SJ, et al. (2009) Predictive value of seizure ICD-9 codes for vaccine safety research. Vaccine 27: 5307–5312. doi:10.1016/j.vaccine.2009.06.092.

37. Shui IM, Rett MD, Weintraub E, Marcy M, Amato AA, et al. (2012) Guillain-Barré syndrome incidence in a large United States cohort (2000-2009). Neuroepidemiology 39: 109–115. doi:10.1159/000339248.

38. Keeney RL, Raiffa H (1976) Decisions with multiple objectives: preferences and value tradeoffs. New York: Wiley. 569 p.

39. Keeney RL (2013) Foundations for Group Decision Analysis. Decision Analysis. Available: http://da.journal.informs.org/content/early/2013/03/13/deca.2013.0265. Accessed 21 August 2013.

40. Keeney RL, Nau R (2011) A theorem for Bayesian group decisions. J Risk Uncertain 43: 1–17. doi:10.1007/s11166-011-9121-5.

41. Bults M, Beaujean DJMA, Richardus JH, van Steenbergen JE, Voeten HACM (2011) Pandemic influenza A (H1N1) vaccination in The Netherlands: parental reasoning underlying child vaccination choices. Vaccine 29: 6226–6235. doi:10.1016/j.vaccine.2011.06.075.
